# The integration of single-cell and metabolomics reveals the increase of oxidative phosphorylation during the liver metastasis of colorectal cancer

**DOI:** 10.1186/s40170-025-00408-z

**Published:** 2025-10-09

**Authors:** Tianyu Liu, Sizheng Sun, Yicheng Huang, Yiming E., Wenyuan Li, Fanggui Xu, Zhengxia Liu, Xiagang Luo, Chen Lu, Chunzhao Yu

**Affiliations:** 1 Department of General Surgery, Sir Run Run Hospital, Nanjing Medical University, Nanjing, Jiangsu 211112 China; 2 Department of General Surgery, The Second Affiliated Hospital of Nanjing Medical University, Nanjing, Jiangsu 210011 China; 3 Department of General Surgery, The Affiliated Taizhou People’s Hospital of Nanjing Medical University, Taizhou, Jiangsu 225300 China; 4 Department of Cardiothoracic Surgery, Zhongda Hospital, School of Medicine, Southeast University, Nanjing, 210009 China; 5 Department of Geriatrics, The Second Affiliated Hospital of Nanjing Medical University, Nanjing, Jiangsu 210011 China; 6 Department of Anesthesiology, The Second Affiliated Hospital of Nanjing Medical University, Nanjing, Jiangsu 210011 China; 7 Department of General Surgery, The Affiliated Suzhou Hospital of Nanjing Medical University, Suzhou, Jiangsu 215002 China

**Keywords:** Colorectal cancer, Liver metastasis, Oxidative phosphorylation, TGFβ

## Abstract

**Background:**

Colorectal cancer (CRC) is among the most prevalent malignant tumors, with liver metastasis as the leading cause of mortality. Although metabolic reprogramming is known to play a crucial role in tumor metastasis, our understanding of this process during colorectal cancer liver metastasis (CRLM) remains limited.

**Methods:**

A stepwise mouse model of CRC liver metastasis was developed, and metabolomic profiling was performed to verify model stability and identify metabolic changes. Single-cell RNA sequencing (scRNA-seq) was used to assess oxidative phosphorylation (OXPHOS) activity within the metastatic tumor microenvironment (TME). Additionally, spatial transcriptomics (ST) was conducted to elucidate the spatial distribution of metabolic phenotypes within metastatic sites. Finally, in vivo experiments were conducted by administering TGFβ inhibitor (LY2157299) or OXPHOS inhibitor (IACS-010759) to evaluate the potential for liver metastasis, and in vitro, the functions of HCT116 and SW620 cells were assessed through Transwell assays and oxygen consumption rate (OCR) measurements.

**Results:**

Metabolomic profiling revealed heightened tricarboxylic acid (TCA) cycle activity in liver metastases. ScRNA-seq analysis showed increased OXPHOS in metastatic cells, including a highly malignant cell subtype characterized by augmented OXPHOS. Further analysis identified a significant upregulation of OXPHOS associated with TGFβ pathway activation. ST localized this OXPHOS -enriched subtype within metastatic tissue. Both in vivo and in vitro experiments demonstrate that inhibition of TGFβ signaling reduces OXPHOS activity, thereby attenuating the progression of colorectal cancer liver metastasis.

**Conclusions:**

This study identifies OXPHOS upregulation as a key metabolic alteration during CRC liver metastasis, which could be induced by TGFβ signaling pathway. These findings contribute to a refined understanding of CRC metabolic adaptation in liver metastases and may inform therapeutic strategies targeting OXPHOS in advanced CRC.

**Supplementary Information:**

The online version contains supplementary material available at 10.1186/s40170-025-00408-z.

## Introduction

Colorectal cancer (CRC) is one of the most common malignant tumors of the digestive system, ranking third in incidence and second in mortality worldwide [1]. The liver is the primary target organ for hematogenous metastasis of CRC, and liver metastasis is the leading cause of death in CRC patients. Among newly diagnosed cases, advanced CRC accounts for approximately 60%, with 22% of patients presenting with distant metastasis [2]. The median survival of untreated patients with colorectal liver metastases is only 6–9 months [3]. Although the median survival can exceed 40 months following combined hepatic resection, most patients still experience recurrence post-surgery [4]. Therefore, colorectal liver metastasis is not only a critical focus but also a major challenge in CRC treatment, and it serves as a key factor in assessing patient prognosis.

Metabolic reprogramming of cancer cells is recognized as one of the hallmarks of cancer [5]. In recent years, accumulating evidence suggests that metabolic reprogramming plays a pivotal role in cancer metastasis [6–8]. Tumor tissues undergo metabolic reprogramming to meet the demands of rapid tumor growth for bioenergetics, biosynthesis, and redox balance [9]. As the central organelle of cellular metabolism, mitochondria play an essential role in cancer metabolic reprogramming [10]. In recent years, various drugs targeting mitochondrial metabolic reprogramming have been under continuous development [11].

In this study, we developed a stepwise model of colorectal liver metastasis and used metabolomics sequencing to uncover the metabolic reprogramming of liver metastases. Furthermore, we validated the metabolic alterations in liver metastases using single-cell and ST, identifying that the TGF-β pathway may reshape mitochondrial oxidative phosphorylation (OXPHOS) in metastatic tumor cells during CRC metastasis. In vivo and in vitro experiments demonstrate that TGFβ inhibitors reduce OXPHOS activity, thereby attenuating the progression of colorectal cancer liver metastasis. This discovery provides a potential therapeutic target for inhibiting colorectal liver metastasis by targeting mitochondrial metabolic reprogramming.

## Materials & methods

### Cell lines and cell culture

The cell lines HCT116 (#FH0027) and SW620 (#FH0021) were purchased from FuHeng Biotechnology Co., LTD in Shanghai, China. All cell lines were authenticated by short tandem repeat (STR) analysis. The STR report were shown in Supplementary Files. The obtained cell lines were maintained according to the provided guidelines. These cell cultures were preserved in a humidified atmosphere containing 5% CO₂ at 37 °C.

### Establishment of a mouse liver metastasis model

All animal experiments were approved by the Institutional Animal Care and Use Committee (IACUC) of Nanjing Medical University (Approval No. IACUC-2008045). The procedures were conducted in accordance with the guidelines of the IACUC. Four-week-old female BALB/c nude mice were selected for the animal study.

Logarithmic-phase HCT116 and SW620 cells were prepared into a cell suspension at a concentration of 5 × 10⁷ cells/mL, and a mouse model of colorectal liver metastasis was established using the intrasplenic injection method. The procedure was as follows: mice were anesthetized with an intraperitoneal injection of 1.25% avertin solution at a dose of 0.1 mL/10 g, immobilized, and their surgical field was disinfected. A small oblique incision (0.5 cm–1.0 cm) was made below the left costal margin, and the abdominal cavity was opened layer by layer. The spleen was Gently exteriorized using tissue forceps, and a 1 mL syringe with a 5-gauge needle was used to inject the cell suspension slowly under the splenic capsule, with approximately 0.2 mL injected per mouse over a period of 3 min. After injection, the splenic capsule became visibly swollen and pale. A sterile cotton swab soaked in 75% alcohol was used to compress the injection site for 2–3 min to stop bleeding and eliminate any cancer cells that may have leaked, preventing peritoneal implantation. The wound was sutured, and the entire procedure adhered strictly to sterile surgical principles. Postoperatively, the mice were returned to a specific pathogen-free (SPF) barrier facility for housing.

On the 30th day post-surgery, the mice were euthanized, and their liver metastases were assessed. Liver metastasis tissues were collected, and colorectal liver metastatic cells (CRLMC) were isolated and cultured. Using the same method, second and third-generation mouse models were established.

### In vivo bioluminescence imaging

For the in vivo bioluminescence imaging, we utilized HCT116 and SW620 cell lines stably expressing the luciferase gene. After a two-week tumor formation period post-modeling, D-Luciferin Potassium Salt (dissolved in PBS buffer at a stock concentration of 15 mg/mL) was administered via intraperitoneal injection at a dose of 150 mg/kg body weight. Following injection, mice were allowed to rest for 10–15 min to ensure thorough systemic distribution of the substrate. Prior to imaging, animals were anesthetized in an induction chamber with 2%–3% isoflurane and subsequently transferred to the imaging system’s heated stage (maintained at 37 °C) in a supine position, where anesthesia was sustained with 1%–1.5% isoflurane delivered via a nose cone. Imaging acquisition parameters were set with exposure times automatically adjusted between 1 and 60 s based on signal intensity. Bioluminescence signals were quantified using Living Image software by defining regions of interest (ROIs) either manually or automatically on superimposed visible and bioluminescent images. The radiant efficiency (measured as radiance, in units of p/s/cm^2^/sr) within each ROI was recorded for subsequent quantitative analysis.

### Isolation of CRLMC

In a biosafety cabinet, the collected liver metastasis tissues were transferred into a culture dish. The tissues were washed three times with PBS containing antibiotics, and connective tissues, blood, and other impurities were removed. The cleaned tumor tissues were transferred to an EP tube, minced, and washed again with PBS containing antibiotics. After centrifugation at 1,000 rpm for 5 min at 25 °C, the supernatant was discarded. The pellet was resuspended in a complete medium containing 1% Type IV collagenase and 0.05% hyaluronidase and placed in a 37 °C shaking incubator at 150 rpm for enzymatic digestion. The digestion process was monitored under a microscope every 30 min, and when the tissue fragments became translucent and fluffy, the solution was filtered through a 70 μm cell strainer. The filtrate was collected and centrifuged at 1,000 rpm for 5 min at 4 °C, the supernatant was discarded, and the cell pellet was resuspended in a complete medium containing antibiotics. The cells were then transferred to a 37 °C incubator with 5% CO₂ for further culture, with media changes and passaging typically performed after approximately 48 h, depending on the condition of the cells.

Each generation of CRLMC isolated from the stepwise liver metastasis model was designated as P0, P1, and P2, where "P" represents primary culture, and the numbers denote the passage generation.

### Transwell assays

Cell migration and invasion assays were performed using 6.5 mm Transwell chambers with 8.0 μm pore polycarbonate membrane inserts (#CLS3422, Corning Inc.). For the migration assay, 750 μL of medium containing 10% fetal bovine serum (FBS) was added to the lower chamber, and then 200 μL of serum-free cell suspension (8 × 10^4^ cells/mL) was seeded into the upper chamber. The cells were incubated at 37 °C for at least 48 h, followed by fixation with 4% paraformaldehyde and staining with crystal violet solution. The stained cells in the upper chamber were gently removed, and the cells that migrated to the underside of the membrane were imaged and counted under a light microscope.

For the invasion assay, before seeding the cells into the upper chamber, the membrane of the upper chamber was coated with 50 μL of Matrigel (diluted 1:10, #356,234, Corning Inc.). The remaining steps were identical to those of the migration assay.

### Hematoxylin and eosin (H&E) staining of formalin-fixed, paraffin-embedded tissues

After euthanizing the mice, the liver tissues were immediately rinsed with pre-cooled PBS to remove any blood. The tissues were then blotted dry with clean filter paper and fixed in 4% paraformaldehyde for more than 24 h. Once fixation was complete, the tissues were sequentially dehydrated in a graded series of ethanol solutions, starting from lower to higher concentrations, to gradually remove water from the tissue blocks. The ethanol in the tissues was then replaced with xylene, and the tissues were embedded in paraffin. The paraffin blocks were sectioned into 4 μm-thick slices. For H&E staining, the sections were first deparaffinized, followed by rehydration. They were then stained with hematoxylin for 5 min and eosin for 2 min.

### Western blot

Cells were lysed using RIPA buffer containing 1% PMSF and phosphatase inhibitors to extract the proteins. Protein concentrations in each group were determined using a BCA protein assay kit (Beyotime, China). Equal amounts of protein (approximately 30 μg) were loaded onto 10% SDS-PAGE for electrophoresis. After electrophoresis, the proteins were transferred onto PVDF membranes, which were blocked with 5% BSA solution at room temperature for 2 h. The membranes were then incubated overnight on a horizontal shaker at 4 °C with the following primary antibodies: anti-ZEB1 (CST, #3396, 1:2000), anti-E-cadherin (1:1000, Abcam, ab40772), anti-N-cadherin (1:1000, Abcam, ab76011), anti-Vimentin (1:1000, Abcam, ab20346), anti-TWIST (CST, #90,445, 1:2000), anti-Snail (CST, #3879, 1:2000)and anti-β-actin (1:5000, Sigma Aldrich #A5441). After incubation with the corresponding secondary antibodies for 1.5 h, protein detection was performed using a chemiluminescent imaging system.

### Extraction and detection of metabolites in liver metastases for metabolomics analysis

After euthanizing the mice, liver tissues from three Generations of mice were collected. The collected liver metastasis tissues and normal liver tissues were ground in liquid nitrogen, and 100 mg of each tissue was used for analysis. For larger tumor tissues, the extra portion was homogenized and subdivided to ensure consistent sampling. Each sample was placed in an EP tube and resuspended in 500 μL of 80% methanol solution, followed by ice bath incubation for 5 min. The samples were then centrifuged at 15,000 g for 20 min at 4 °C in a refrigerated centrifuge. The supernatant was diluted with mass spectrometry-grade water to reduce the methanol content to 53%, followed by another round of centrifugation under the same conditions. The supernatant was collected and analyzed under the following mass spectrometry conditions: spray voltage, 3.5 kV; scan range, m/z 100–1500; auxiliary gas flow rate, 10 L/min; sheath gas flow rate, 35 psi; S-lens RF level, 60; capillary temperature, 320 °C; polarity, both positive and negative modes; auxiliary gas heater temperature, 350 °C. MS/MS secondary scans were performed using data-dependent acquisition (DDA). The chromatographic conditions were as follows: column, Hypesil Gold (C18); column temperature, 40 °C; flow rate, 0.2 mL/min. For positive mode, mobile phase A was 0.1% formic acid and mobile phase B was methanol. For negative mode, mobile phase A was 5 mM ammonium acetate (pH 9.0) and mobile phase B was methanol.

### Metabolomics data processing and analysis

The raw data were processed using CD3.1 software. Initial filtering was performed based on the mass-to-charge ratio (m/z) and other relevant parameters. Peak alignment across different samples was conducted according to retention time deviation and mass deviation (part per million, ppm). Peaks were then extracted based on the specified ppm, signal-to-noise ratio (S/N), and adduct ion information, and peak areas were quantified. Missing values were imputed via k-nearest neighbors. Only metabolites detected in ≥ 80% of samples were retained.

Subsequently, the high-resolution MS/MS spectra were matched against the mzCloud and mzVault databases, as well as the MassList database for primary mass spectra, to identify metabolites and obtain final identification results for further analysis. The metabolomics data were converted using metaX software, and principal component analysis (PCA) was performed. We applied Z-score normalization to standardize the metabolite signal intensities. Specifically, for each metabolite, the mean value (μ) from the normal liver group was used as the reference, and the standard deviation (σ) served as the scaling factor. This transformation converted the data into a standard normal distribution with a mean of 0 and a standard deviation of 1. Statistical significance (*P*-value) of metabolites between comparison groups was calculated using a t-test, and differentially expressed metabolites were selected based on the criteria of logFC > 1.5 and adj.P.Val < 0.05. A volcano plot was generated to visualize these results. Finally, the KEGG database was used to investigate the functions and metabolic pathways of the identified metabolites.

### Mitochondrial membrane potential assay (JC-1 Staining)

For analysis of the mitochondrial membrane potential, the collected cells were stained with the JC-1 fluorescent probe (Beyotime, Nantong, China) and analyzed using the fluorescence microscope.

### Metabolites measurement

Intracellular levels of isocitrate, fumarate, malate, and the NAD⁺/NADH ratio were quantified using commercial assay kits (Beyotime, China) following the manufacturer’s instructions. Briefly, cells were lysed using the corresponding extraction buffers, and the supernatants were collected after centrifugation. Metabolite concentrations were determined by measuring absorbance at specific wavelengths using a microplate reader.

### Data acquisition and processing

The scRNA-seq data (GSE245552), bulk RNA sequencing datasets (GSE143985 and GSE159216), and spatial transcriptomics (ST) data (GSE225857) were retrieved from the Gene Expression Omnibus (GEO) database. The scRNA-seq dataset comprised 39 samples from 16 colorectal cancer (CRC) patients, including 16 primary tumor samples, 4 normal colon samples, 17 colorectal liver metastasis samples, and 2 normal liver samples. Additionally, TCGA-COAD RNA-seq data with corresponding clinical information were obtained from The Cancer Genome Atlas (TCGA) database. For prognostic model development, the TCGA-COAD cohort served as the primary training set, while GSE143985 was designated as the external validation set. The GSE159216 dataset was partitioned into training and validation subsets at a 7:3 ratio to facilitate internal validation. All the clinical annotations associated with the datasets were systematically gathered, and samples with lacking survival data were eliminated for subsequent survival analyses.

### Quality control, batch effect correction, and cell annotation of scRNA-seq data

The raw expression values of the dataset were read using the Read10X function from the Seurat (version 4.4.0) package [12]. We processed the single-cell data and created Seurat objects using the CreateSeuratObject function (with min.cells = 5 and min.features = 300). Cells that met the following criteria were selected for further analysis: (1) the number of detected Genes per cell ranged from 300 to 6000; (2) mitochondrial gene UMIs were < 50%; (3) ribosomal gene UMIs were < 20%; and (4) total UMIs exceeded 1000, with the top 3% of cells with the highest UMIs excluded. After removing low-quality cells, DoubletFinder (version 2.0.4) was used to filter doublets, assuming a doublet rate of 5% [13].

To remove batch effects between samples, we integrated the single-cell data using the Harmony package (version 1.2.0) [14]. Clustering was performed using the FindClusters function with a resolution of 0.4, which divided the data into 22 clusters based on the top 20 principal components. Visualization was achieved using Uniform Manifold Approximation and Projection (UMAP). Marker genes for each cluster were identified using the FindAllMarkers function in Seurat (default parameters), and cell types were annotated into seven major categories based on the CellMarker database (Supplementary Table 3).

### InferCNV analysis

We performed chromosome copy number variation (CNV) analysis on all epithelial cells using the inferCNV package (version 1.3.3) (https://github.com/broadinstitute/inferCNV) [15]. Endothelial cells were used as a reference to calculate the initial CNV score for each epithelial cell. Non-malignant epithelial cells were filtered based on the average CNV score of normal colon epithelial cells.

### Training and verification of the model

A univariate Cox regression analysis was conducted on the differentially expressed genes (DEGs) of the Malignant_14 subgroup to identify genes significantly associated with COAD prognosis (p < 0.05). Subsequently, a LASSO Cox regression model (R package "glmnet") was employed to refine the candidate gene list and construct a prognostic model. The penalty parameter (λ) was determined using the minimum criterion, and the final model retained the selected genes along with their coefficients. The TCGA expression dataset was standardized using the "scale" function in R, and risk scores for each sample were calculated. Based on the median risk score, TCGA-COAD patients were stratified into low-risk and high-risk subgroups, and Kaplan–Meier survival analysis was performed to compare overall survival (OS) between the two groups. The predictive performance of the model was evaluated by Generating ROC curves for 1-year, 3-year, and 5-year survival using the "survival", "survminer", and "timeROC" R packages. To validate the robustness of the model, external validation was conducted using the GSE143985 COAD cohort from the GEO database. For the Malignant_03 subgroup, the same methodology was applied to split the GSE159216 dataset into training and validation sets at a ratio of 7:3 for model training and validation.

### Monocle2 pseudotime and CytoTRACE analysis

We used Monocle (version 2.30.1) to infer the pseudotime trajectory of malignant epithelial cells in the CRC scRNA-seq dataset [16]. A CellDataSet object was created using the UMI count matrix with the negbinomial.size parameter. The dispersionTable function was used to calculate variable genes, and significant genes were selected based on the following criteria: (1) mean expression > 0.1; (2) empirical dispersion > 1 × fitted dispersion. Dimensionality reduction and cell ordering were performed using the DDRTree method and orderCells function.

Cell differentiation trajectories and pseudotime gene expression changes were visualized using the plot_cell_trajectory and plot_genes_branched_pseudotime functions, respectively. Finally, we used the CytoTRACE package (version 0.3.3) to score cell stemness, assigning each cell a score between 0 and 1, with higher scores indicating greater stemness (less differentiation) and lower scores indicating less stemness (more differentiation) [17]. The CytoTRACE scores were projected onto the Monocle pseudotime trajectory for visualization to determine the starting point of the pseudotime trajectory.

### CellChat analysis

To investigate the differences in intercellular communication between epithelial cells with different metabolic profiles, we used the CellChat R package (version 1.6.1) to infer potential intercellular communication networks [18]. Data from primary tumors and liver metastases were imported separately, and CellChat objects were created using the createCellChat function. After preprocessing the expression data using the identifyOverExpressedGenes, identifyOverExpressedInteractions, and projectData functions, the computeCommunProb function was applied to calculate potential ligand-receptor interactions. The filterCommunication function (min.cells = 10) was then used to filter out subtypes with low communication frequency. Finally, communication probabilities for signaling pathways were calculated using the computeCommunProbPathway function, and aggregate intercellular communication networks were generated using the aggregateNet function. The communication networks were compared between the primary tumor (PT) and liver metastasis (LM) groups.

### Hallmark pathway enrichment analysis, GSEA, and GSVA

We performed Hallmark pathway enrichment analysis of differentially expressed genes using gene set enrichment analysis (GSEA) with the clusterProfiler package (version 4.1.0) [19]. The enriched results were visualized using the GseaVis package (version 0.1.0) (https://github.com/junjunlab/GseaVis). Additionally, gene set variation analysis (GSVA) was conducted using the GSVA package (version 1.5.0) to assess pathway activity scores for each cell [20]. Differences in pathway activity between groups were calculated using the limma package (version 3.58.1), with pathways considered differentially active if the absolute t-value exceeded 2 [21]. The enriched pathways were visualized using the ggplot2 package.

### ST data processing

We processed the ST data using the Seurat package, filtering out spots with fewer than 10 detected genes. The UMI counts for the remaining spots were normalized using SCTransform, followed by dimensionality reduction with RunPCA. Clustering was performed using FindNeighbors (dims = 30) and FindClusters (resolution = 0.8), and visualization was achieved using RunUMAP.

Given that each Visium sample spot contains multiple cells, we performed deconvolution of the ST data using the SPOTlight package (version 1.6.7) combined with scRNA-seq data [22]. After filtering mitochondrial and ribosomal Genes, we identified the top 3000 highly variable genes using modelGeneVar. Highly variable genes (HVGs) with an average AUC > 0.8 were selected to predict the major cell types within each spot. The distribution of major cell types in the ST data was visualized using the SpatialFeaturePlot function.

To determine the spatial distribution of the high OXPHOS subtype, we selected the top 20 genes of the high OXPHOS subtype from the scRNA-seq data and calculated gene enrichment scores for each spot using the AddModuleScore function. The differentiation status of cells within each spot was assessed using CytoTRACE. To estimate the metabolic state of each spot, we applied both GSVA and scFEA (version 1.0) on the ST data to infer cellular metabolic states [23]. scFEA performs this inference by reconstructing a comprehensive human metabolic map and applying a probabilistic model and neural network model with flux balance constraints on scRNA-seq data.

We used PROGENy (version 1.24.0) to estimate pathway activity for each spot [24]. PROGENy infers pathway responses in human samples by utilizing data from publicly available perturbation experiments to pinpoint critical changes at pathway nodes, particularly in cancer or diseased tissues. The PROGENy model includes 14 pathways, and we performed the analysis using the top 500 most responsive genes for each pathway. The results for the TGF-β pathway were visualized.

### Measurement of cell OCR

OCR was measured using the Seahorse XFe 96 extracellular flux analyzer (Agilent Technologies) in conjunction with the Seahorse XF Cell Mito Stress Test Kit (product number: 103015–100). Briefly, 2 × 10^4^ CRC cells were seeded into XF96-well cell culture plates and incubated at 37 °C overnight. During the assay, mitochondrial inhibitors were sequentially added at specified time points according to the kit protocol (Agilent Technologies, 103,015–100): first, 1.5 μM oligomycin was introduced to inhibit ATP synthase and assess ATP-linked respiration; subsequently, 1 μM carbonyl cyanide 4-(trifluoromethoxy)phenylhydrazone (FCCP) was added to uncouple mitochondrial respiration and determine the maximal respiratory capacity; finally, 0.5 μM rotenone/antimycin A (Rot/Anti-A) was applied to block the electron transport chain and measure non-mitochondrial respiration. The assay was performed strictly following the manufacturer's instructions, and data were analyzed using Seahorse Wave software. Following the measurement, cells from each well were trypsinized and counted to normalize the OCR data. For quantitative analysis, non-mitochondrial OCR values were subtracted to reflect mitochondrial respiration accurately.

### Statistical analysis

All statistical analyses and data computations in this study were conducted using R (version 4.3.2), Python (version 3.7), and GraphPad Prism 9.0. Data following a normal distribution are presented as mean ± standard deviation, while data with a skewed distribution are expressed as median values. Statistical tests used in this study include the student's t-test, Wilcoxon rank-sum test, and Pearson correlation analysis. Differences were considered statistically significant if *P* < 0.05 (ns, *p* ≥ 0.05; *, *p* < 0.05; **, *p* < 0.01; ***, *p* < 0.001). Each experiment was independently repeated at least three times.

## Results

### The liver metastatic potential of CRC lines gradually increases in the stepwise liver metastasis model

**Fig. 1:**
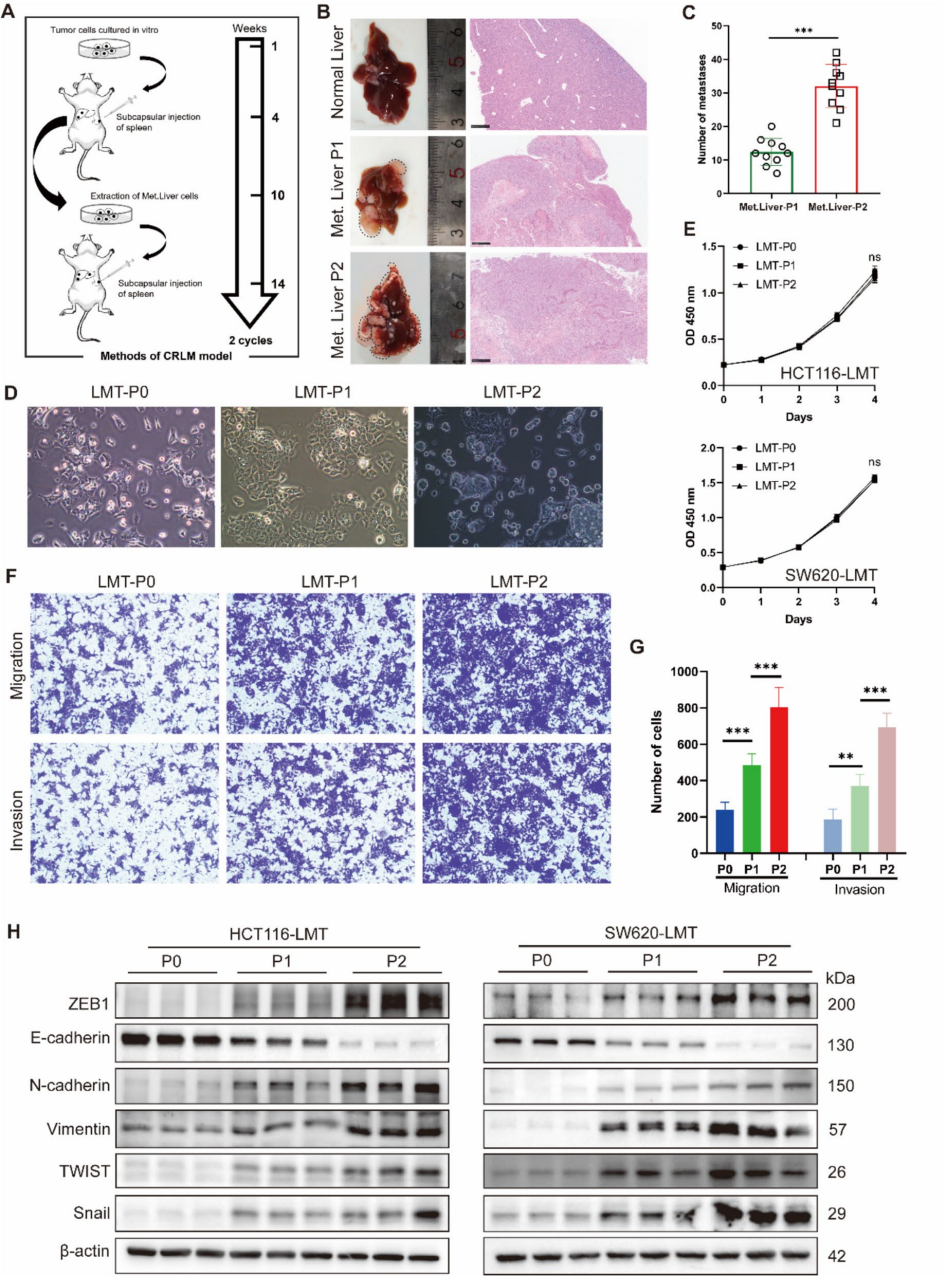
A stepwise model of liver metastasis in mice with CRC. **A** Graphical overview of the design of a ladder model of liver metastasis in CRC. **B**, **C** Collect representative photos and H&E-stained slides of each group of liver metastases, and record the number of metastatic lesions. **D** The morphologies of liver metastatic cells under a 20 × microscope. **E** The CCK8 assay evaluated the proliferation of P0, P1, and P2 generations of HCT116 and SW620 cells. **F**, **G** The Transwell migration assay assessed the migratory and invasive capabilities of cells in each group. Scale bar: 100 µm. *p < 0.05, **p < 0.01, and ***p < 0.001, Student's t-test. **H** Western blotting experiments demonstrated the expression alterations of key proteins in the EMT signaling pathways

We successfully established a human CRC liver metastasis stepwise model in mice using the SW620 and HCT116 cell lines (Fig. 1A), with the SW620 cell line model having been previously reported [25]. Upon examining liver metastasis in the HCT116 cell line model, we observed that compared to the original HCT116 cells (P0), the number of liver metastatic foci in the first generation (P1, *n* = 10) and the second generation (P2, *n* = 10) significantly increased, showing a progressive upward trend (Fig. 1B-C). Morphological analysis revealed no significant changes in CRLMC P1 and P2 cells compared to the parental cell line (Fig. 1D). However, when these cells were removed from the liver metastasis microenvironment, their migratory capacity began to decline significantly after an average of more than six passages (Supplementary Fig. 1). The CCK8 showed no significant differences in proliferative activity among the P0, P1, and P2 generations of both HCT116 and SW620 cells (Fig. 1E). Transwell assays demonstrated that the migratory and invasive capacities of CRLMC cells increased with each generation (P < 0.05, Fig. 1F-G). Similarly, the expression levels of epithelial-mesenchymal transition (EMT)-related markers, including ZEB1, N-cadherin, Vimentin, TWIST and Snail gradually increased, while E-cadherin expression significantly decreased(Fig. 1H). These results suggest that the metastatic potential of the tumor cells increases with successive passaging.

### Metabolomics sequencing reveals metabolic reprogramming in liver metastases

**Fig. 2:**
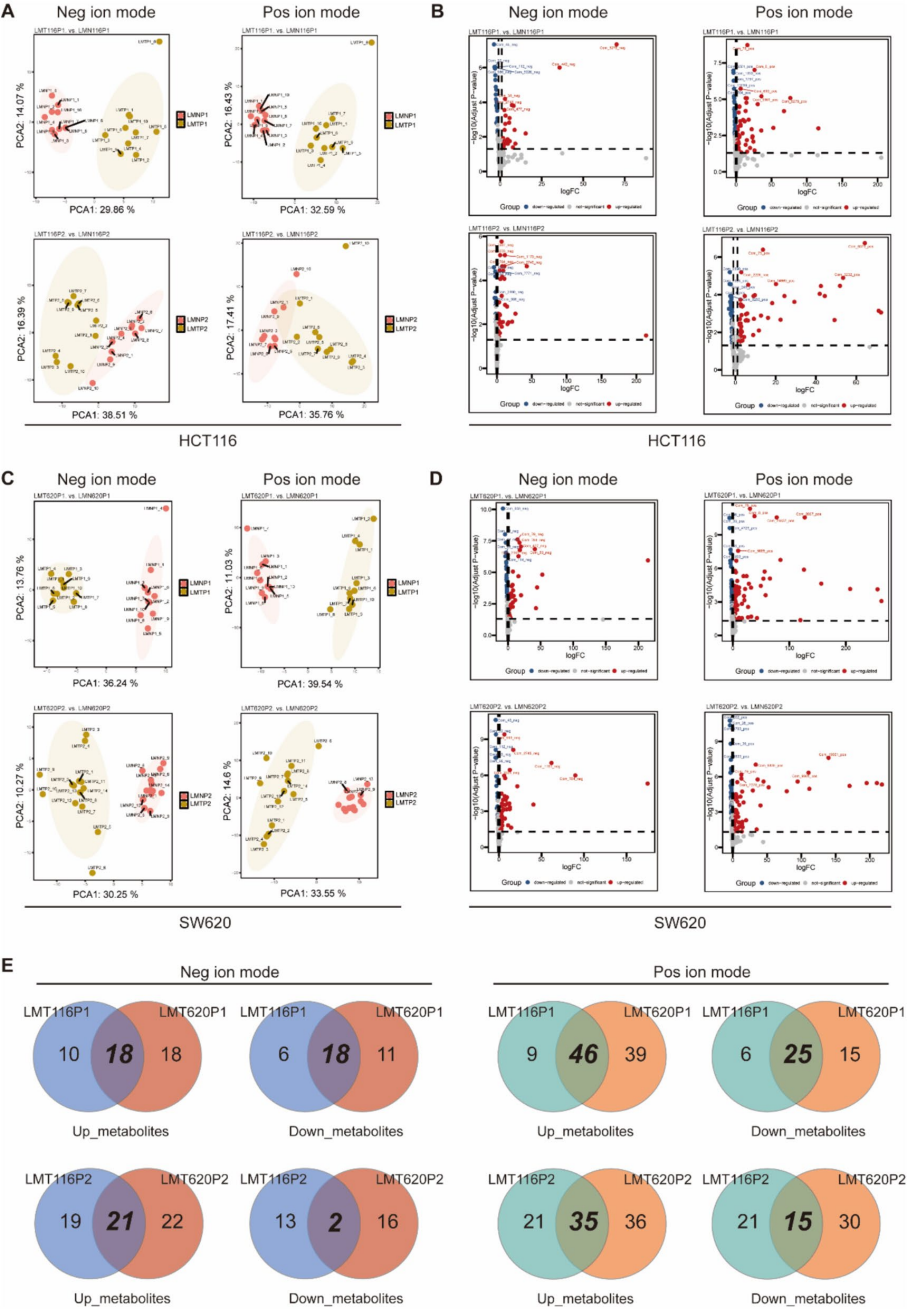
Metabolomics reveals that liver metastases possess distinct metabolic patterns. **A**, **C** PCA of positive and negative ion metabolites in liver metastases derived from HCT116 (**A**)and SW620(**C**) cell lines as compared with normal liver. Scatter points of different colors indicate the samples of different experimental groups, and the ellipses represent the 95% confidence interval (When the number of biological replicates is less than 4, the 95% confidence ellipse cannot be presented). **B** The volcano plot of differential metabolites screened from the comparison group of LMT116 and LMN116 derived from P1 and P2. **D** The volcano plot of differential metabolites screened from the comparison group of LMT620 and LMN620 derived from P1 and P2. **E** The positive ion metabolites and negative ion metabolites that coexist in the HCT116 and SW620 cell lines in either P1 or P2

To explore the metabolic reprogramming in liver metastasis cells across different generations, we performed untargeted metabolomics sequencing on three generations of liver metastases using LC–MS/MS technology. Given the high dimensionality and strong correlation among variables in metabolomics data, we applied principal component analysis (PCA) for further analysis. The results demonstrated significant differences in both positive and negative ion mode metabolites between liver metastasis cells and normal liver cells within the same generations of HCT116 and SW620 cell lines. However, the metabolic profiles of cells within each group remained consistent (Fig. 2A, C). This not only suggests that liver metastasis cells undergo metabolic reprogramming but also confirms the stability of the liver metastasis model.

We set the thresholds for differential metabolites at logFC > 1.5 and adj.P.Val < 0.05, and displayed the differential metabolites between liver metastasis cells (P1 and P2) and normal liver cells in the HCT116 and SW620 cell lines using volcano plots (Fig. 2B, D). Among the identified metabolites, several were commonly present in both cell lines (Fig. 2E). In negative ion mode, 18 upregulated and 18 downregulated metabolites were shared between the two cell lines in P1, while 21 upregulated and 2 downregulated metabolites were shared in P2. In positive ion mode, 46 upregulated and 25 downregulated metabolites were shared in P1, while 35 upregulated and 15 downregulated metabolites were shared in P2. These differential metabolites are listed in Supplementary Table 1 and Supplementary Table 2.

We then performed KEGG pathway enrichment analysis for the shared metabolic profiles in each generation. The results showed that, compared with normal liver tissues, the TCA cycle was significantly enriched in first-generation liver metastatic cells (Supplementary Fig. 2 A), but no significant enrichment was observed in the second generation (Supplementary Fig. 2B). This suggests that as metastatic potential strengthens, TCA cycle activity gradually increases to a level comparable to that of normal liver tissues. Overall, liver metastatic cells exhibit metabolic characteristics distinct from those of normal liver tissues; however, TCA cycle activity progressively escalates with successive generations.

### The stepwise liver metastasis model reveals the enhanced mitochondrial function in metastatic tumor cells

**Fig. 3:**
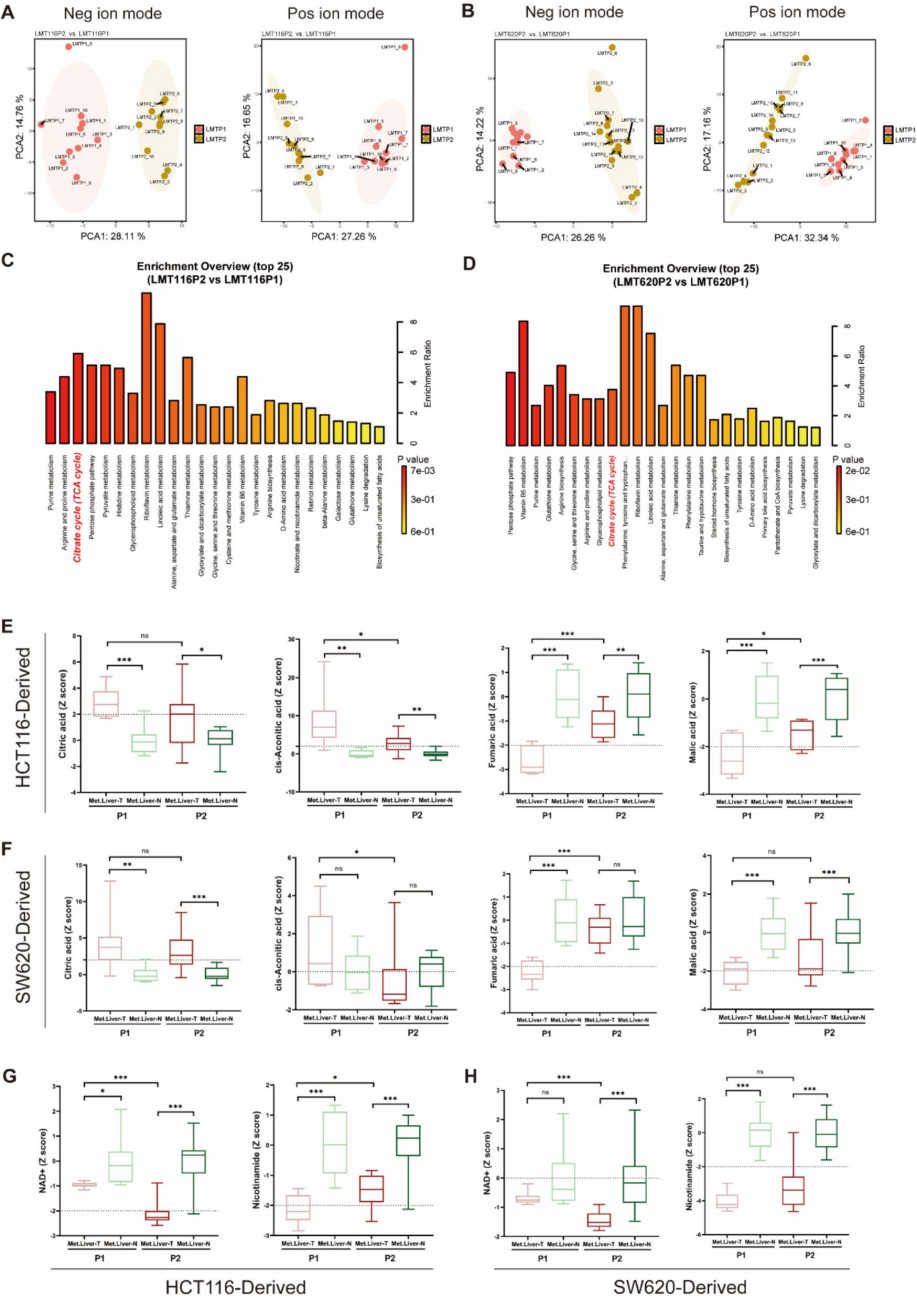
Metabolomics uncovers the enhanced of the TCA cycle in liver metastases. **A** PCA of the positive and negative ion metabolites of LMT116P2 and LMT116P1. **B** PCA of the positive and negative ion metabolites of LMT620P2 and LMT620P1. **C** KEGG pathway enrichment of the positive and negative ion metabolites of LMT116P2 and LMT116P1. **D** KEGG pathway enrichment of the positive and negative ion metabolites of LMT620P2 and LMT620P1. **E** Metabolic differences in citric acid, cis-aconitic acid, fumaric acid, and malic acid in HCT116 cell line. **F** Metabolic differences in citric acid, cis-aconitic acid, fumaric acid, and malic acid in SW620 cell line. **G** Discrepancies in the metabolism of NAD + and nicotinamide within the HCT116 cell line. **H** Discrepancies in the metabolism of NAD + and nicotinamide within the SW620 cell line

To investigate the metabolic changes in metastatic tumors during multiple passages, we found significant differences in both positive and negative ion mode metabolites between different generations (P1 and P2) of CRLM models constructed by HCT116 and SW620 cell lines (Fig. 3A, B). This suggests that the metabolic profile of liver metastasis cells is continuously reshaped as their invasive capacity increases. We then performed KEGG enrichment analysis on the differential metabolites between P1 and P2 for both cell lines, revealing that the TCA cycle was significantly enriched in both groups (Fig. 3C, D). For comparative purposes, we normalized each group of data using Z-score with data of normal liver tissues as the baseline respectively. The results showed that in both the HCT116 and SW620 cell lines, as the generations progressed, citrate and aconitate were gradually depleted, while fumarate and malate accumulated (Fig. 3E, F). This indicates that the TCA cycle in liver metastasis cells was reactivated. Concurrently, NAD + was increasingly utilized, with the production of its precursor NMN also rising (Fig. 3G, H), suggesting that the mitochondrial OXPHOS function was gradually enhanced compared to normal liver.

Subsequently, we quantitatively analyzed the relative levels of isocitric acid, fumaric acid, malic acid and the NAD +/NADH ratio in the P0, P1, and P2 generations of HCT116 and SW620 cells. The results revealed that the levels of isocitric acid and the NAD +/NADH ratio progressively decreased, whereas the levels of Fumaric acid and Malic acid progressively increased (Supplementary Fig. 3 A, B). Additionally, we used JC-1 staining to assess changes in mitochondrial membrane potential across generations and found a significant increase in mitochondrial membrane potential in P2 cells, indicating marked mitochondrial activation (Supplementary Fig. 3 C, D).

In summary, as the invasive capacity of metastatic tumors increases, mitochondrial OXPHOS function is progressively enhanced.

### scRNA-Seq identifies high OXPHOS subtypes in CRC

**Fig. 4:**
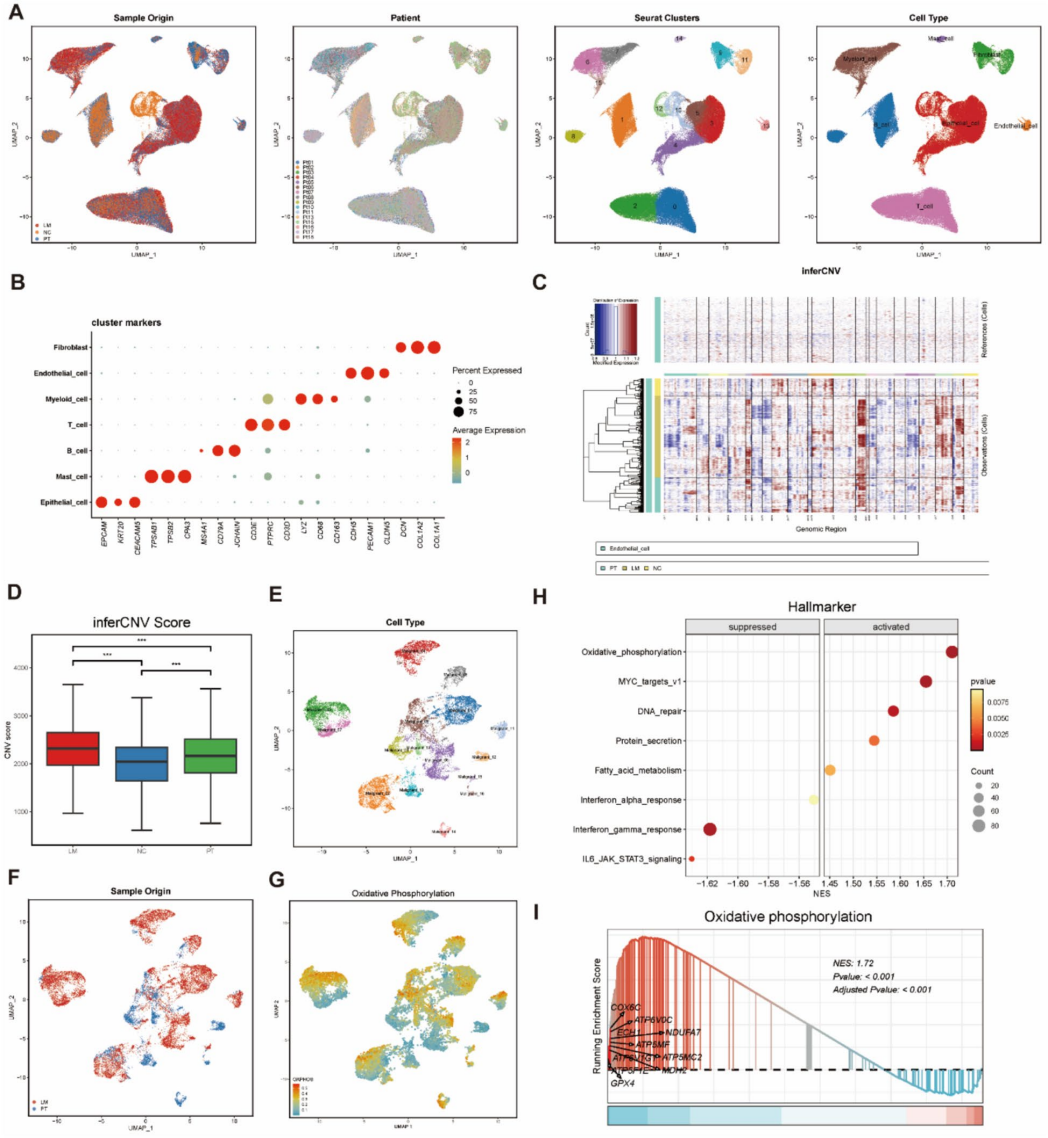
Single-cell transcriptomics atlas of paired primary CRC and liver metastasis. **A** UMAP plots of scRNA-seq profiled in this study were colored based on sample origin, patient, cluster and cell type. **B** Bubble map representing the expression of marker genes in major cell types. **C** Chromosomal heat maps of inferred CNVs in epithelial cells, with red denoting amplification and blue signifying deletion. **D** Boxplot for CNV Scores (NC, PT and LM). *p < 0.05, **p < 0.01, and ***p < 0.001, Student's t-test. **E** UMAP plots of malignant epithelial cells, with 16 clusters. **F** The UMAP map colored based on sample origin(PT and LM). **G** The OXPHOS scoring map of all malignant epithelial. **H** Bubble plot depicting the hallmarks of DEGs between LM and PT. The intensity represents the *p*-values, and the dot size reflects the gene count for each hallmark. **I** The GSEA enrichment score of OXPHOS and the top 10 genes

To further explore the changes in OXPHOS during CRC metastasis, we downloaded publicly available single-cell RNA sequencing data from the GeO database. After performing quality control, filtering out doublets, and correcting for batch effects, we retained 37 samples from 16 patients for further analysis, resulting in 134,136 single-cell transcriptomes (Supplementary Fig. 4 C, D). Using UMAP clustering, we divided these cells into 16 clusters and annotated seven major cell types based on classical cell marker genes (Supplementary Fig. 4 A, B), including T cells, B cells, epithelial cells, myeloid cells, fibroblasts, endothelial cells, and mast cells (Fig. 4A, B).

Given that normal and malignant epithelial cells in CRC exhibit distinct metabolic profiles, we used endothelial cells as reference cells to infer copy number variations (CNVs) in all epithelial cells (Fig. 4C). As expected, the CNV levels in the liver metastasis (LM) group were significantly higher than in the primary tumor (PT) group, while the normal colon (NC) group exhibited the lowest CNV levels (Fig. 4D). Using the average CNV score of NC epithelial cells as a threshold, we classified epithelial cells into malignant (19,769 cells) and non-malignant (18,955 cells) types. Malignant epithelial cells were then reclustered into 16 clusters, and visualization was performed using UMAP (Fig. 4E, F).

Next, we applied the scMetabolism package to score OXPHOS activity in malignant epithelial cells and projected these scores onto the UMAP for visualization (Fig. 4G). The results indicated that high OXPHOS cells were predominantly distributed in the LM group. Gene set enrichment analysis (GSEA) of the LM and PT groups revealed significant enrichment of the OXPHOS pathway in the LM group (Fig. 4H, Supplementary Fig. 4E), and we visualized the trend of enrichment scores and the top 10 genes (Fig. 4I). Further analysis of OXPHOS scores identified Malignant_03 and Malignant_14 as the two clusters with the highest OXPHOS activity, originating from the LM and PT groups, respectively (Supplementary Fig. 4 F). Finally, we examined differentially enriched pathways in GO and KEGG terms between Malignant_03 and Malignant_14(Supplementary Fig. 5). Our findings indicated that Malignant_14 was significantly enriched in pathways associated with adaptive immune response, immunoglobulin complex, natural killer cell-mediated cytotoxicity, and chemokine signaling, suggesting a stronger capacity for immune cell recruitment in primary tumors. In contrast, Malignant_03 showed predominant enrichment in pathways related to protein folding, the cell cycle, glycolysis/gluconeogenesis, and the MAPK signaling pathway, indicating increased proliferative activity and heightened energy metabolism demands in liver metastases.

In conclusion, we found that malignant epithelial cells in the LM group exhibited higher OXPHOS levels, and a high OXPHOS subtype was identified in both the PT and LM groups.

### Constructing a risk score model for the high OXPHOS level subtype of CRC

**Fig. 5:**
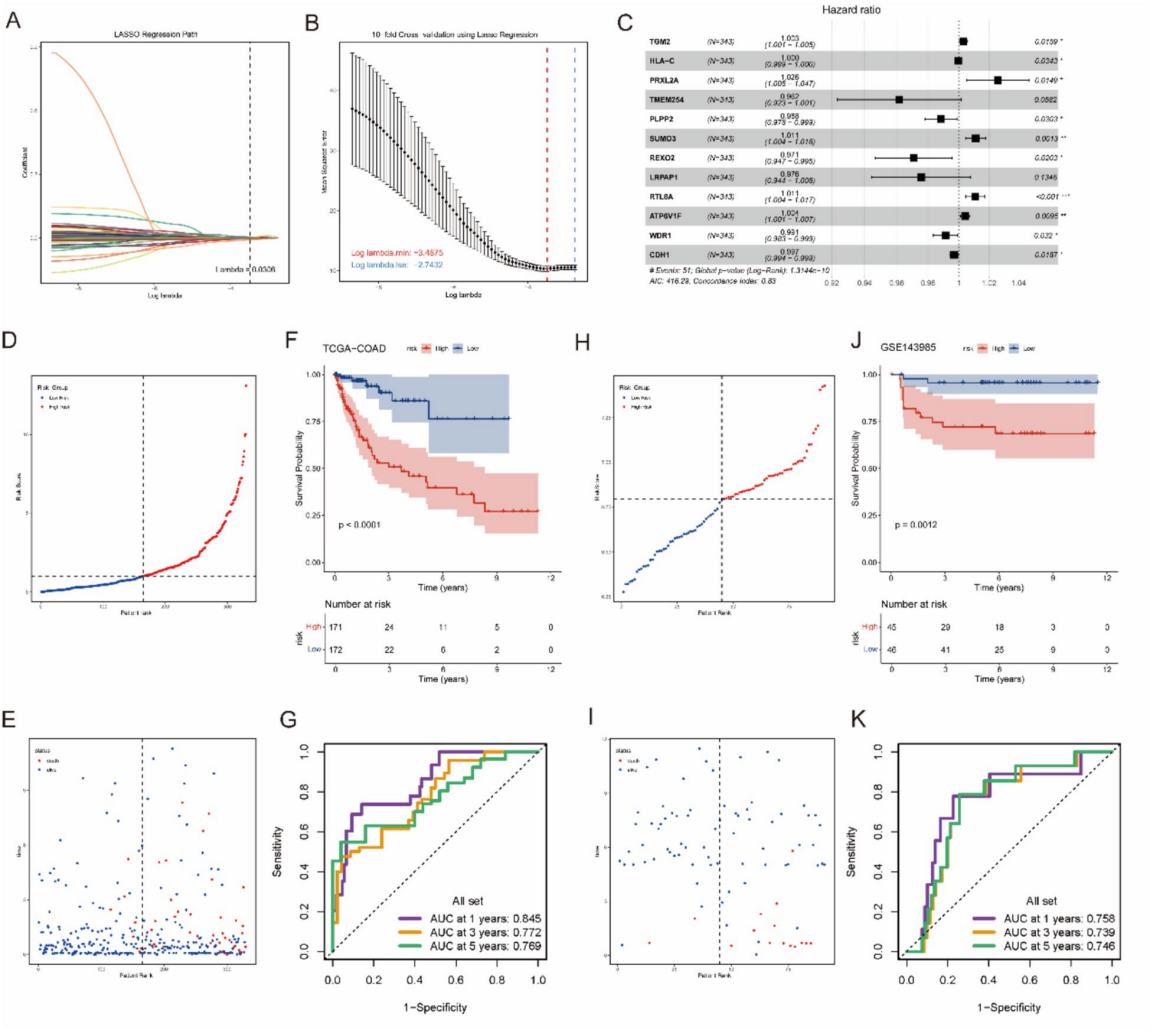
The model was constructed and validated using the differential genes from the Malignant_03. **A**, **B** Ten-fold cross-validation determined the penalty regularization parameter λ for gene selection. **C**. Multivariate Cox regression analysis of the genes in the model. **D**, **E**. The risk score and corresponding survival status of samples from the TCGA-COAD dataset. **F**. The relationship between the risk score of samples in the TCGA-COAD dataset and OS. **G**. The relationship between risk scores and ROC curves of samples from the TCGA-COAD dataset. **H**, **I** The risk score and survival status of the ascending sorted samples in the GSE143985 dataset. **J**. The relationship between the risk score of samples in the GSE143985 dataset and OS. **K**. The relationship between risk scores and ROC curves of samples from the GSE143985 dataset

To evaluate the prognostic risk of the subgroup with high oxidative phosphorylation levels in colorectal cancer, we constructed risk scoring models for the Malignant_03 and Malignant_14 subgroups. We used the TCGA-COAD dataset as the training set and the GSE143985 dataset for validation. Univariate Cox regression analysis was performed on the differentially expressed genes of the Malignant_14 subgroup, identifying 72 genes significantly associated with colorectal cancer prognosis. In the LASSO analysis, tenfold cross-validation was applied to determine the penalty regularization parameter λ, which was set at 0.0306 (Fig. 5A, B). Based on this, a multivariate Cox regression analysis was conducted using the bidirectional stepwise regression method to select 12 genes as factors. The final model was constructed using the risk coefficients of each factor (Fig. 5C). The formula for the risk score is as follows: Risk score = 0.0030TGM2—0.0004HLA-C + 0.0255PRXL2A—0.0392TMEM254—0.0118PLPP2 + 0.0109SUMO3—0.0296REXO2—0.0247LRPAP1 + 0.0107RTL8A + 0.0040ATP6V1F—0.0088WDR1—0.0032CDH1.

The risk scores for the TCGA-COAD and GSE143985 dataset samples were subsequently calculated using this model and ranked by risk score in ascending order (Fig. 5D, E, H, I). For the TCGA-COAD dataset, the survival curves of high-risk samples (*n* = 171) and low-risk samples (*n* = 172) showed starkly different trends (Fig. 5F). The survival curve of high-risk samples exhibited a sharp decline, while the curve for low-risk samples remained relatively flat, staying above 75%. The area under the ROC curve (AUC) for 1-year, 3-year, and 5-year overall survival (OS) was 0.845, 0.772, and 0.769, respectively (Fig. 5G). These results indicate that the model provides ideal survival predictions for patients in the TCGA-COAD dataset. The survival trends between high-risk (*n* = 45) and low-risk (*n* = 46) samples in the GSE143985 dataset also showed significant differences, with the death risk for high-risk samples consistently higher than for low-risk samples (Fig. 5J). Furthermore, the model accurately predicted the 1-year, 3-year, and 5-year OS in the GSE44295 dataset, with AUC values consistently above 0.7 (Fig. 5K).

We applied the same approach to construct a risk score model for the Malignant_03 subgroup, using the GSE159216 dataset, which was split into a training set and a validation set in a 7:3 ratio. The model demonstrated ideal predictive performance (Supplementary Fig. 6).

### Relationship between risk scores and immune checkpoint molecule expression

**Fig. 6:**
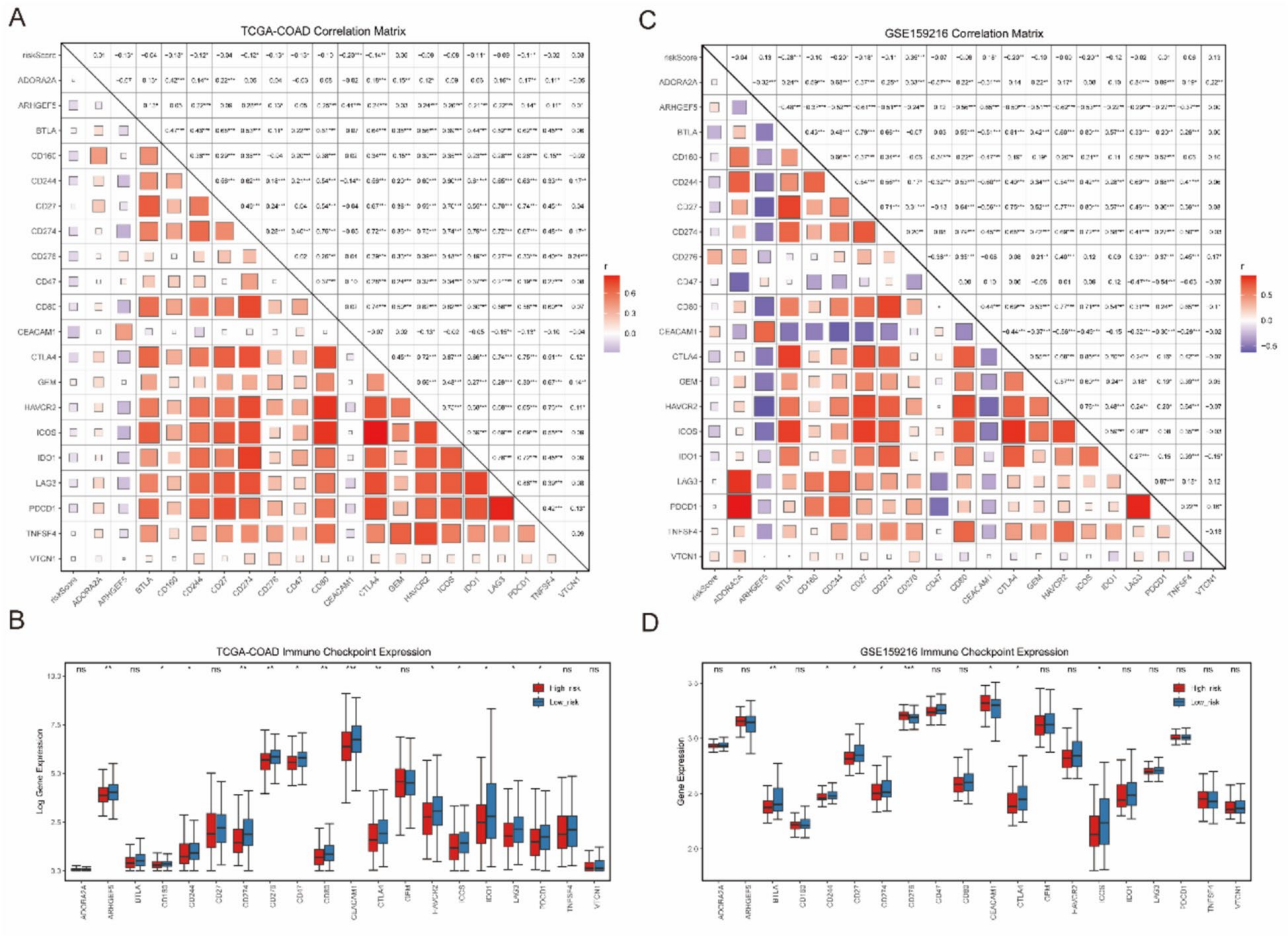
The association between risk scores and immune checkpoint expression levels. **A**, **C** Correlation analysis between immune checkpoint expression from HisgAtlas and risk scores in the TCGA-COAD and GSE159216 dataset. **B**, **D** Box plots of immune checkpoint expression in high-risk and low-risk TCGA-COAD and GSE159216 samples from HisgAtlas

Tumors evade anti-tumor immunity by upregulating immune checkpoint ligands and recruiting immune-suppressor cells, resulting in an immunosuppressive tumor microenvironment. Tumors with a highly immunosuppressive microenvironment are linked to impaired immune cell cytotoxicity and more aggressive behavior, leading to poor prognosis. We retrieved 20 immune checkpoint molecules from the HisgAtlas and constructed heatmaps to examine the correlation between their expression and risk scores in both the TCGA-COAD cohort and the GSE159216 dataset. In the TCGA-COAD cohort, the expression of 10 immune checkpoint molecules was negatively correlated with risk scores (Fig. 6A). Of the 20 immune checkpoints tested, 14 showed significant expression differences between low-risk and high-risk samples, all of which were significantly downregulated in the high-risk group (Fig. 6B). In the GSE159216 dataset, the expression of CD276 and CEACAM1 was positively correlated with risk scores, while the expression of the remaining six molecules was negatively correlated (Fig. 6C). Among the 20 immune checkpoints tested, only 8 showed significant expression differences between low-risk and high-risk samples, with 6 of them being significantly downregulated in the high-risk group (Fig. 6D). In conclusion, compared to primary lesions, liver metastases show differential expression of immune checkpoints, indicating that they may possess a distinct immune evasion capability.

### Monocle2 reveals the shift from glycolysis to OXPHOS in malignant glandular epithelial cells

**Fig. 7:**
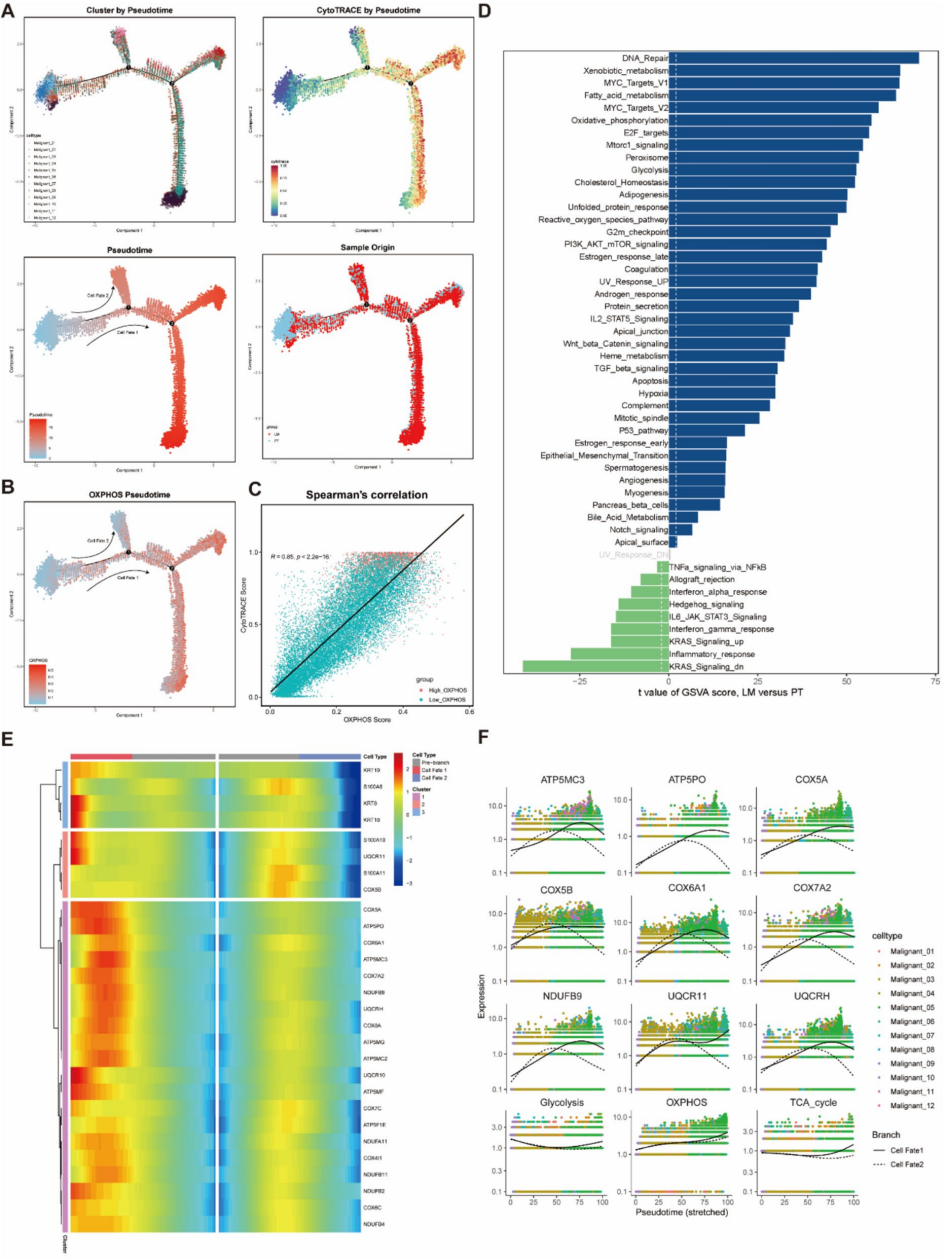
Identifying the characteristics of high OXPHOS epithelial cells via pseudotime analysis. **A** The potential trajectories of all malignant epithelial cells are separately colored by cell type, CytoTRACE score, pseudo-time, and sample origin. The arrows indicate the possible direction of epithelial cell differentiation. **B** The pseudotime plot of OXPHOS score. **C** The Spearman correlation between the CytoTRACE score and the OXPHOS score. **D** High OXPHOS epithelial cells and other malignant epithelial cells differ in the pathway activity of each cell as measured by GSVA scores. **E** The heatmap below shows the relative changes in gene expression from low to high, with colors ranging from blue to red, for differentially expressed genes between cellfate1 and cellfate2 after the epithelial cell clusters were classified into three based on the pseudotime clustering results. **F** Dotplot of the glycolysis and OXPHOS pathways and key genes based on the KEGG database

To dissect the evolutionary dynamics of CRC glandular epithelial cells, we performed pseudotime analysis on 16 glandular epithelial clusters and constructed a developmental trajectory (Supplementary Fig. 7 A). This trajectory featured four branches, outlining the developmental pathway from malignant epithelial cells to liver metastasis epithelial cells. By combining CytoTRACE scores to assess differentiation potential, we identified that the Malignant_12 cluster, which exhibited the lowest differentiation potential, was located at the left side of the trajectory, confirming this as the starting point of the developmental pathway (Fig. 7A).

Next, we projected the OXPHOS scores onto the trajectory (Fig. 7B, Supplementary Fig. 7B) and observed a bifurcation near branch point 1, leading to two fates: Cell Fate 1, with high OXPHOS levels, and Cell Fate 2, with low OXPHOS levels. The clusters with the highest OXPHOS levels, Malignant_03 and Malignant_14, were mainly located within Cell Fate 1 (Supplementary Fig. 7 A). More importantly, we found a significant positive correlation between OXPHOS and CytoTRACE scores in malignant glandular epithelial cells (Fig. 7C), suggesting that OXPHOS may play a crucial role in the liver metastasis of CRC.

Previous work by Liu et al. emphasized the importance of OXPHOS in the metastatic process of CRC, and our findings are consistent with theirs [26]. We performed enrichment analysis using Hallmark gene sets from the Molecular Signatures Database (MsigDB) (Fig. 7D), with the most significant enrichment found in DNA repair, xenobiotic metabolism, MYC signaling, and OXPHOS, aligning with the clinical pathological features of patients and the increased malignancy in the high OXPHOS subtype. Interestingly, we observed that hepatic metastases exhibited concurrent activation of OXPHOS alongside other metabolic pathways, including fatty acid metabolism, glycolysis, and cholesterol homeostasis, all of which are closely associated with tumor progression [27–29]. This reflects the high plasticity of metabolic reprogramming in cancer cells during metastasis. Of particular note was the simultaneous enhancement of both glycolysis and OXPHOS, which may be attributable to the spatial heterogeneity of the tumor microenvironment. Liver metastases typically comprise hypoxic regions, such as areas distant from blood vessels, where cancer cells primarily rely on glycolysis for survival, and oxygen-rich regions adjacent to hepatic sinusoids, where OXPHOS is preferentially activated [30, 31]. This functional partitioning within the metastatic niche enables the entire lesion to demonstrate an overall upregulation of both glycolytic and oxidative metabolism. As a result, hepatic metastases exhibit elevated ATP production capacity and enhanced biosynthetic potential, supporting their aggressive growth and survival.

Next, we conducted BEAM analysis, with a heatmap displaying the driver Genes during the transition from the initial state to Cell Fate 1 and Cell Fate 2 (Fig. 7E, Supplementary Fig. 7 C), most of which were related to OXPHOS. In the pseudotime trajectory, key OXPHOS genes (ATP5MC2, COX6C, NDUFB4, UQCR10) exhibited an upward trend in Cell Fate 1, while showing an initial increase followed by a decrease in Cell Fate 2 (Fig. 7F, Supplementary Fig. 7D). In contrast, glycolysis showed a downward trend followed by an upward trend in both Cell Fate 1 and Cell Fate 2 (Fig. 7F). This indicates a metabolic shift in malignant glandular epithelial cells from glycolysis to OXPHOS.

In conclusion, our findings reveal the evolutionary shift between OXPHOS and glycolysis during CRLM, suggesting that OXPHOS may play a pivotal role in the metastatic process.

### Intercellular interactions of the high OXPHOS subtype in the TME

**Fig. 8:**
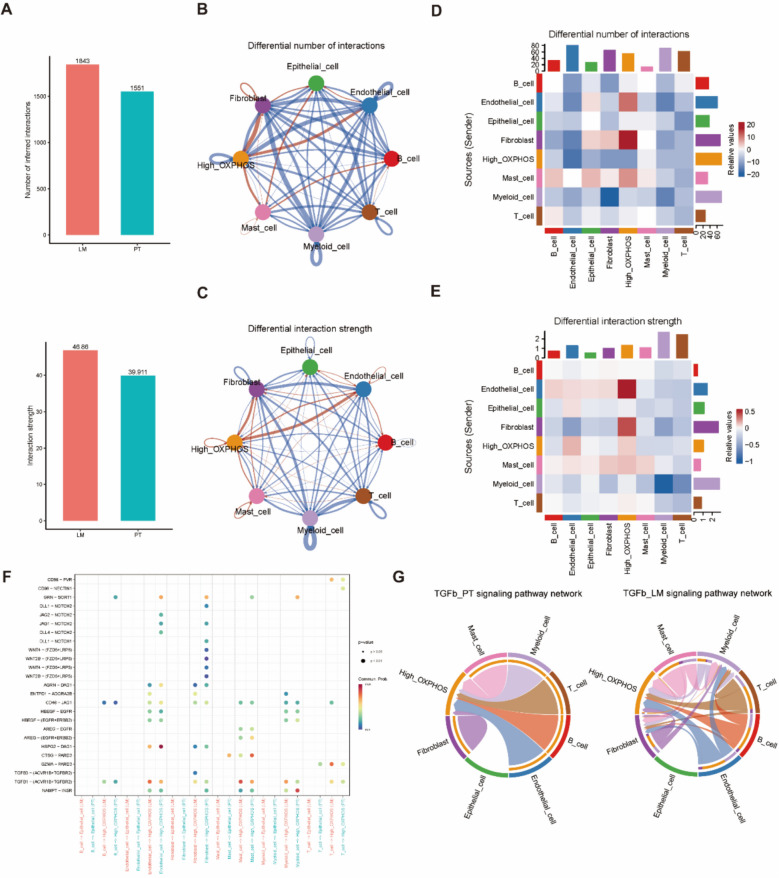
CellChat reveals high OXPHOS epithelial with cell-to-cell ligand-receptor interactions in the TME. **A** The bar plot shows the number and strength of intercellular interactions in PT and LM. **B**, **C** The plot of the number (**B**) and strength (**C**) of intercellular interactions, where the size of the points is proportional to the number of cells and the thickness of the lines is proportional to the number or strength of the interactions. The arrows extend from the ligand cells to the receptor cells. **D**, **E** The heatmap reveals the variance in the number (**D**) and strength (**E**) of intercellular interactions between PT and LM. **F** The bubble plot illustrates the communication status of the high OXPHOS epithelial specific ligand-receptors within the PT and LM. The size of the dots indicates the *P* value, the color of the dots represents the probability of communication, and the blank areas signify a communication probability of zero. **G** Chord diagrams portraying the inferred TGF-β signal network in PT (Left) and LM (Right)

To further investigate the regulatory signaling pathway changes of the high OXPHOS subtype within the tumor microenvironment (TME), we applied the CellChat tool to analyze intercellular communication. The results indicated that although the number and strength of interactions among the major cell types in the LM group were higher than in the PT group (Fig. 8A), the differences were relatively small (Fig. 8B, C). In the LM group, the high OXPHOS subtype exhibited stronger interactions with endothelial cells and fibroblasts, while interactions with immune cells were significantly weaker compared to the PT group (Fig. 8D, E). This suggests that malignant epithelial cells in the LM group possess enhanced immune evasion capabilities and a greater ability to remodel the TME.

Subsequently, we compared the specific ligand-receptor interactions between the high OXPHOS subtype and other epithelial cells in both the PT and LM groups. We identified five signaling pathways uniquely expressed in the high OXPHOS subtype (Fig. 8F), including VISFATIN, TGF-β, HSPG, EGF, and AGRN (Fig. 8G). VISFATIN enhances tumor cell invasiveness by activating the PI3K/Akt and MAPK signaling pathways and concurrently drives metabolic reprogramming in high OXPHOS subtype cells to meet the energy demands of metastasis [26]. TGF-β, in advanced stages, induces epithelial-mesenchymal transition (EMT) to disrupt cell adhesion, facilitating tumor cell detachment from the primary lesion and entry into the circulatory system [32, 33]. HSPG remodels the extracellular matrix by binding growth factors and regulates cell migration and invasion signals within the tumor microenvironment [34]. EGF activates the MAPK/ERK and PI3K/Akt pathways via EGFR to promote proliferation and enhances ATP generation through CHD6-mediated mitochondrial oxidative phosphorylation [35]. AGRN activates the WNT signaling pathway via the Lrp4 receptor, strengthening the matrix adhesion and migration capabilities of tumor cells, and its specific expression can assist in identifying metastatic foci [36, 37]. These pathways form a network through mechanisms such as metabolic regulation, signal transduction, and microenvironment remodeling, collectively driving the metastatic cascade, with TGF-β activation being the most extensive. In conclusion, the CellChat analysis revealed that several unique pathways are activated in the high OXPHOS subtype within the TME.

### ST combined with scRNA-Seq reveals the spatial characteristics of the high OXPHOS subtype

**Fig. 9:**
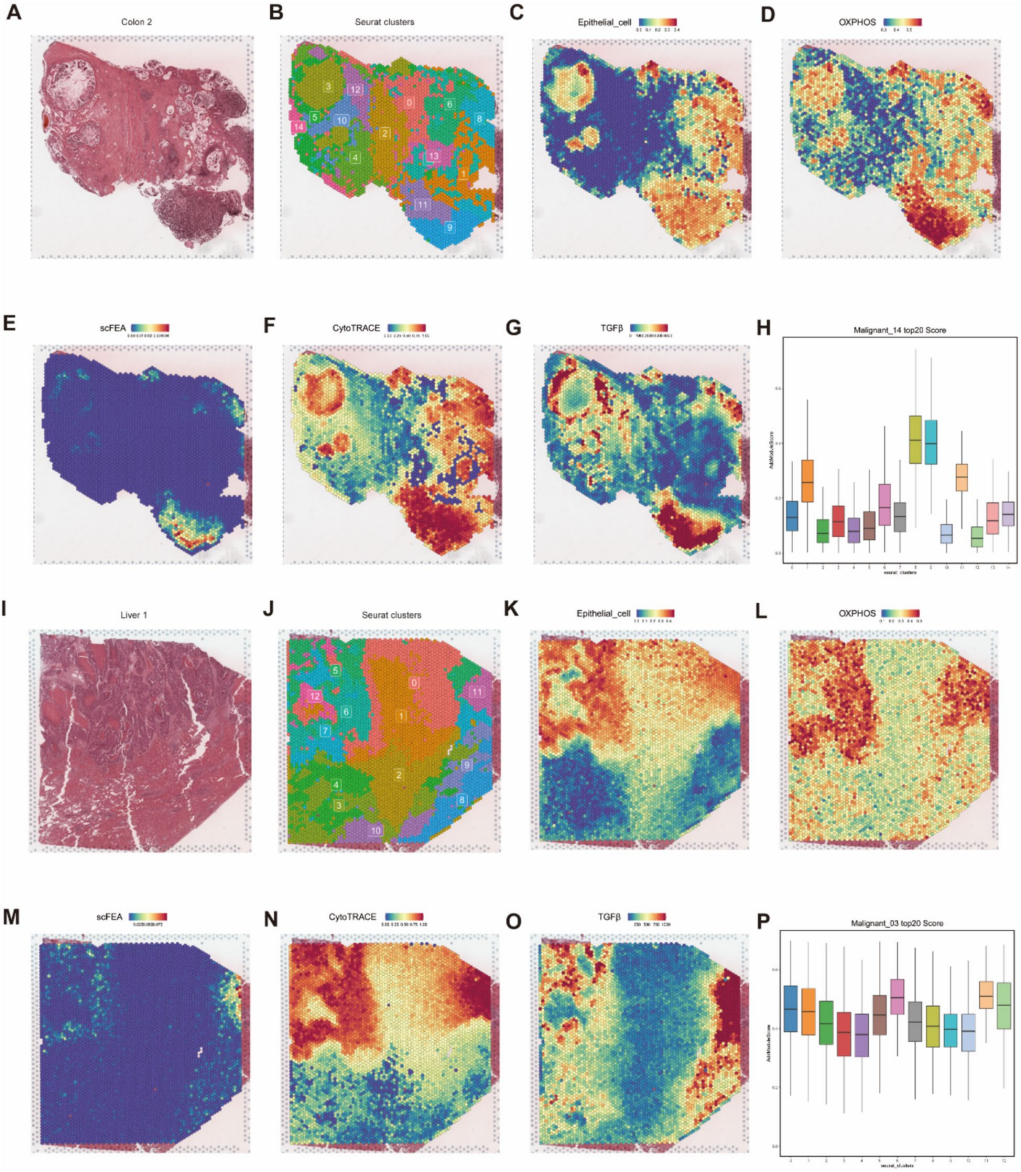
ST atlas of High OXPHOS epithelial. **A**, **I** H&E staining of tissue sections from PT(A) and LM(I). **B**, **J** The cluster distribution map obtained after clustering the ST subclusters. **C**, **K** The spatial distribution map of epithelial cells predicted by SPOTlight. **D**, **L** All ST spots of OXPHOS scores by GSVA. **E**, **M** Metabolic flux perturbation from succinate to fumarate within the spatial context. **F**, **N** The spatial mapping of cellular stemness scores predicted by CytoTRACE. **G**, **O** Spatial mapping of the TGF-β pathway score predicted by PROGENy. **H**, **P** The barplot depicting the scores assigned to the top 20 genes within the high OXPHOS subcluster by using AddModuleScore

To further validate the spatial distribution characteristics of the high OXPHOS subtype, we utilized ST data from CRC patients available in the GeO database. Using the Seurat package, we divided one of the primary tumor samples into 0–14 subgroups (Fig. 9A, B). SPOTlight was then applied to deconvolute the ST data based on scRNA-seq data, and we visualized the spatial distribution of epithelial cells (Fig. 9C). By scoring the top 20 genes from Malignant_14 using the AddModuleScore function, we found that subgroups 8, 9, and 11 had the strongest correlation with Malignant_14 (Fig. 9H).

Next, we performed GSVA to assess the OXPHOS levels in the ST data, revealing that subgroups 8, 9, and 11 had the highest OXPHOS scores (Fig. 9D). Single-cell flux estimation analysis, using a graph-based neural network model, estimated the metabolic flux rate for each spatial spot in the ST data (Fig. 9E). CytoTRACE was used to assess the differentiation status of the epithelial cells (Fig. 9F). The results showed that subgroups 8, 9, and 11 not only exhibited higher malignancy but also had a significant increase in TCA cycle activity.

To precisely pinpoint key pathway changes in the ST data, we used PROGENy, which revealed significant enrichment of the TGF-β pathway in subgroups 8, 9, and 11 (Fig. 9G), confirming the invasion process of the high OXPHOS epithelial subtype at the spatial level.

We conducted the same analysis on another liver metastasis sample to validate these findings, dividing the ST data into 0–12 subgroups (Fig. 9I, J), and performed deconvolution using SPOTlight (Fig. 9K). We found that subgroups 6 and 11 had the strongest correlation with Malignant_03 (Fig. 9P). GSVA OXPHOS scores, scFEA TCA metabolic flux scores, CytoTRACE scores, and TGF-β pathway activity were all significantly enriched in subgroups 6 and 11 (Fig. 9L), which was consistent with the results from the first sample.

In conclusion, we validated at the spatial level that the high OXPHOS epithelial subtype possesses strong invasive potential, and this process may be driven by the activation of the TGF-β signaling pathway.

### Inhibition of TGFβ suppresses OXPHOS activity and attenuates colorectal liver metastasis progression

**Fig. 10:**
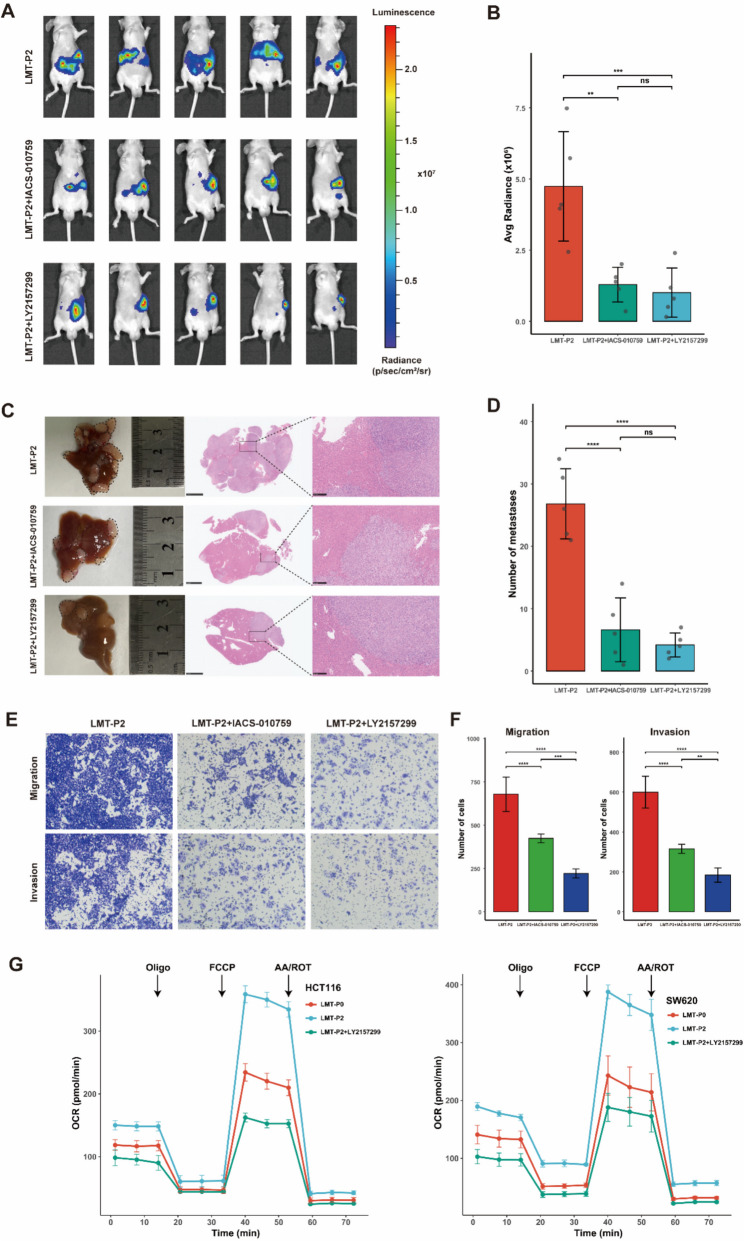
LY2157299 inhibits CRC metastasis and OXPHOS activity. **A**, **B** Bioluminescence images and fluorescence quantification bar charts from mice treated with LY2157299 and IACS-010759 compared to the control group. **C**, **D** Representative photographs and H&E stained sections of liver metastases from both the experimental and control groups, along with the recorded number of metastatic lesions. **E** Transwell assay comparing the migratory capacity of HCT116-LMT-P2 and HCT116 cells treated with LY2157299 or IACS-010759. **F** Bar chart summarizing the cell counts from the three Transwell assays. **G** OCR profiles were monitored in the indicated HCT116-LMT-P2 and SW620-LMT-P2 cells using a Seahorse XFe 96 analyzer. Metabolic inhibitors were injected at specific time points, as indicated. Data are presented as mean values ± SD from three biologically independent experiments

To elucidate the regulatory mechanism between TGFβ and OXPHOS, we performed both in vivo and in vitro experimental validations. In the in vivo study, a liver metastasis model was established in nude mice by subcapsular splenic injection of luciferase-expressing HCT116-LMT-P2 cells. The animals were randomly assigned to three groups: LMT-P2 cells (control group), LMT-P2 + TGFβ inhibitor (LY2157299), and LMT-P2 + OXPHOS inhibitor (IACS-010759). Bioluminescence imaging demonstrated that both LY2157299 and IACS-010759 significantly reduced the luminescent signal from liver metastases (Fig. 10A, B). Histopathological analysis using HE staining and metastatic lesion counting further revealed that OXPHOS inhibition significantly reduced the number of hepatic metastases (Fig. 10C, D). In vitro Transwell assays under the same treatment conditions showed that both inhibitors significantly attenuated the migratory and invasive capacities of HCT116 cells (Fig. 10E, F). Furthermore, Oxygen consumption rate (OCR) assays in HCT116 and SW620 cell lines demonstrated that OXPHOS metabolism was significantly elevated in LMT-P2 cells compared to LMT-P0 cells, and this elevation was markedly reduced upon LY2157299 treatment (Fig. 10G). Collectively, these findings indicate that TGFβ inhibition suppresses liver metastasis in colorectal cancer, potentially through the downregulation of OXPHOS metabolism.

## Discussion

In this study, we established a stepwise liver metastasis model of CRC in mice and used metabolomics sequencing to identify changes in TCA metabolism in liver metastases. We further identified a subtype characterized by high OXPHOS from paired CRC primary and liver metastasis lesions. By integrating scRNA-seq and ST data, we characterized this subtype at both spatial and temporal levels. During the liver metastasis of CRC, this subtype experiences the reactivation of the TCA cycle and a significant enhancement of OXPHOS.

Metabolic reprogramming is a critical aspect of cancer cell reprogramming. Research on cancer metabolism reprogramming dates back to the 1920 s, when Otto Warburg first reported that tumor tissues metabolize more glucose and produce more lactate than healthy tissues, a phenomenon known as the Warburg effect [38]. However, recent studies have increasingly shown that many cancers rely on OXPHOS for survival and proliferation [39–41]. In CRC, it has been demonstrated that an increase in mitochondrial DNA copy number enhances the metastasis of microsatellite-stable CRC cells, a mechanism driven by elevated mitochondrial OXPHOS [42]. Additionally, enhanced OXPHOS is a hallmark of cancer stem cells, and inhibiting OXPHOS in CRC stem cells can suppress their metastatic potential [26, 43]. High OXPHOS activity may maintain the stemness characteristics of tumors through multi-dimensional mechanisms in synergy. Firstly, mitochondrial metabolic intermediates driven by OXPHOS, such as acetyl-CoA, regulate epigenetic modifications like histone acetylation, thereby activating the expression of core stem cell genes such as OCT4 and NANOG [44, 45]. Secondly, moderate reactive oxygen species (ROS) generated by OXPHOS activate pro-survival signaling pathways such as PI3K/AKT and Wnt/β-catenin, enhancing the self-renewal ability and therapeutic resistance of cancer cells [46]. Notably, OXPHOS-dependent CRC stem cells demonstrate metabolic adaptability in nutrient-limited microenvironments via efficient ATP generation, providing an energy advantage that supports their survival under hypoxia or chemotherapy pressure [11, 47]. Furthermore, the stability of the mitochondrial dynamic network, regulated by proteins such as MFN2 and DRP1, is closely associated with the maintenance of stemness. High OXPHOS activity may enhance the stem cell phenotype through PGC1α-mediated mitochondrial biogenesis [47, 48]. These discoveries systematically elaborate the molecular framework through which OXPHOS maintains tumor stemness via the metabolic-epigenetic-signaling network, laying a foundation for the development of precise therapeutics targeting OXPHOS and mitochondrial dynamics.

The divergent TCA cycle activity between primary colorectal tumors (suppressed) and liver metastases (activated) underscores metabolic plasticity as a hallmark of cancer progression. This shift likely arises from adaptive responses to microenvironmental, transcriptional, and biosynthetic demands. The oxygen- and nutrient-rich hepatic microenvironment contrasts sharply with the hypoxic, resource-limited primary tumor niche. In the liver, ample oxygen destabilizes HIF-1α—a glycolysis promoter—thereby relieving its suppression of mitochondrial respiration. Meanwhile, abundant glucose, glutamine, and lipids directly fuel the TCA cycle [49, 50]. Metastatic cells further amplify TCA flux by upregulating key enzymes such as pyruvate carboxylase (PC) and isocitrate dehydrogenase (IDH), which replenish cycle intermediates (e.g., oxaloacetate) and generate α-ketoglutarate to sustain proliferation [51]. Enhanced TCA activity not only meets the energetic demands of metastasis but also supplies biosynthetic precursors (citrate, succinyl-CoA) and redox cofactors (NADH, NADPH) critical for macromolecule synthesis and oxidative stress resistance [52, 53]. This metabolic reprogramming reflects a strategic adaptation: primary tumors prioritize glycolysis for survival under hypoxia, whereas metastases exploit the liver’s resources to reactivate mitochondrial metabolism, aligning with their aggressive proliferative phenotype. These findings highlight the TCA cycle as a context-dependent vulnerability, suggesting that targeting anaplerotic enzymes (e.g., PC) or nutrient uptake pathways in metastases could disrupt their metabolic fitness. However, the regulatory interplay between host-tumor crosstalk, epigenetic modifications, and systemic metabolism in driving this shift warrants deeper exploration. Elucidating whether TCA cycle activation is a driver or consequence of metastatic colonization may unlock novel therapeutic strategies to impede metabolic adaptation during cancer dissemination.

Our findings demonstrate that colorectal cancer (CRC) liver metastasis cells exhibit markedly elevated oxidative phosphorylation (OXPHOS) activity, which correlates with enhanced invasive capacity in vitro. Mechanistically, heightened OXPHOS likely fuels metastasis by sustaining energy demands through efficient ATP production, thereby supporting critical processes for invasion such as cytoskeletal remodeling, matrix degradation, and pseudopod formation [54, 55]. Notably, while moderate mitochondrial ROS generated via OXPHOS may activate pro-metastatic pathways (e.g., HIF-1α, NF-κB), CRC cells counteract excessive ROS toxicity by upregulating antioxidant systems [56]. Furthermore, OXPHOS activity confers survival advantages to circulating tumor cells by maintaining mitochondrial membrane potential and anti-apoptotic BCL-2 family protein expression, thereby resisting anoikis during dissemination [57]. Intriguingly, the metabolic flexibility of OXPHOS, including the utilization of alternative substrates like glutamine or fatty acids, facilitates adaptation to nutrient-poor metastatic niches [58]. These observations align with reports linking mitochondrial electron transport chain inhibition to suppressed metastasis in CRC [59], but contrast with studies emphasizing glycolysis in metastatic progression [60–63]. Such discrepancies may reflect dynamic metabolic shifts during distinct stages of metastasis, wherein OXPHOS predominates in later phases, such as colonization. Our data underscore OXPHOS as a potential therapeutic target in metastatic CRC, though further studies are warranted to delineate the context-dependent interplay between glycolysis and mitochondrial metabolism.

In our study, the KEGG analyses in Supplementary Figs. 2 and Figs. 3 C-D both demonstrated the activation of multiple metabolic pathways. In the enrichment analysis of Supplementary Fig. 2, the results revealed that both arginine biosynthesis and arginine and proline metabolism pathways were significantly enriched in liver metastatic tissues compared with normal liver tissues in both the P1 and P2 generations. Several previous studies have confirmed that inhibiting arginine metabolism significantly suppresses colorectal cancer liver metastasis, which is consistent with our analytical findings [64–66]. Given the central role of the TCA cycle in cellular metabolism, arginine can be converted via the urea cycle to glutamate, which subsequently enters the TCA cycle, thereby providing additional metabolic support for tumor cells. In the enrichment analysis shown in Fig. 3 C-D, we found that purine metabolism, arginine and proline metabolism, TCA cycle, pentose phosphate metabolism and other pathways were significantly enriched between the two generations. These pathways are all closely related to liver metastasis of colorectal cancer. However, considering that the TCA cycle is located at the core hub of cell metabolism, its dysfunction plays a driving role in liver metastasis of colorectal cancer. This reprogramming of the TCA cycle may, in conjunction with the active purine metabolism, meet the biosynthetic demands, interact with the alteration of arginine metabolism to influence the immune microenvironment, and cooperate with the enhancement of the pentose phosphate pathway to maintain redox homeostasis [67–69]. Therefore, we selected the TCA cycle and OXPHOS as the central metabolic pathways for in-depth analysis.

This study has several limitations. First, the sample size of the animal models remains limited, posing a risk of sample bias. The liver metastasis model used spleen injection rather than colonic mucosal injection, which does not fully mimic the most pathologically relevant liver metastasis route. Second, the heterogeneity of tumor cells across different patients makes it difficult to identify common characteristics from single-cell sequencing data. Finally, the single-cell and ST data were not derived from the same patient, which limits the robustness of our ST validation.

## Supplementary Information

Supplementary Material 1.

Supplementary Material 2.

Supplementary Material 3.

Supplementary Material 4.

## Data Availability

The scRNA-seq data, bulk RNA-seq data and ST data were both derived from the GEO database, and the accession numbers were GSE245552, GSE143985, GSE159216 and GSE225857 respectively. The datasets analysed during the current study can be provided by the corresponding author if you make a reasonable request.
