# Supplementary material for: The integration of single-cell and metabolomics reveals the increase of oxidative phosphorylation during the liver metastasis of colorectal cancer
Source: Cancer Metab. 2025 Oct 9;13:41. doi: 10.1186/s40170-025-00408-z (PMC12512483; doi:10.1186/s40170-025-00408-z)
Supplement: Supplementary file 1 — Supplementary Material 1. [file 40170_2025_408_MOESM1_ESM.docx]

**
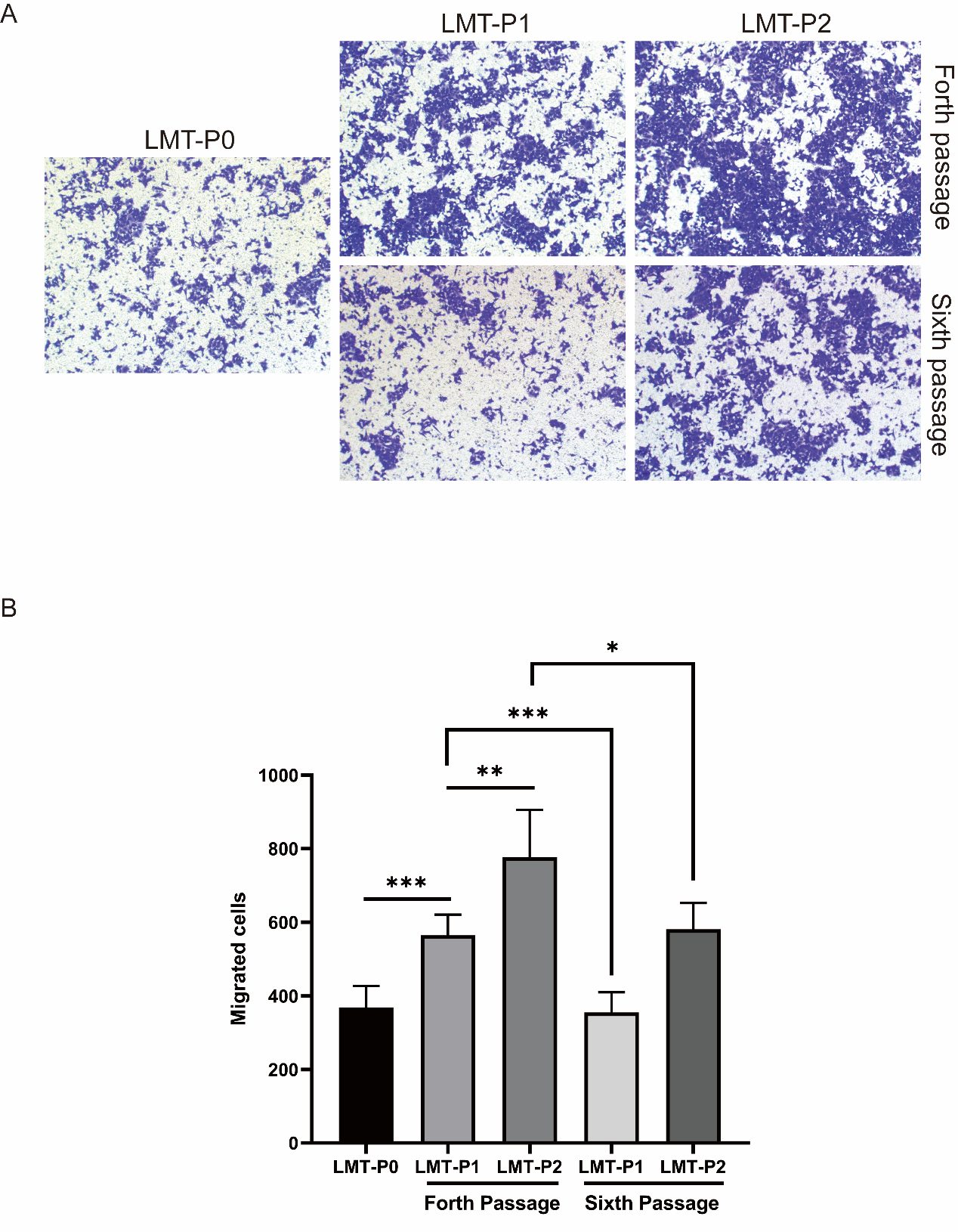
**

**Supplementary Figure 1. The migration capability of HCT116-LMT cells begins to show a significant decline after an average of more than 6 passages.**(A) Transwell migration assay assessed the migratory capability of cells in LMT-P0, LMT-P1 (The Forth passage and Sixth passage) and LMT-P2 (The Forth passage and Sixth passage) groups. These cells are derived from the HCT116 cell line. Scale bar: 100µm.

(B) Bar plot of migarted cells in each group. *<0.05, **p<0.01, ***p<0.001.

**
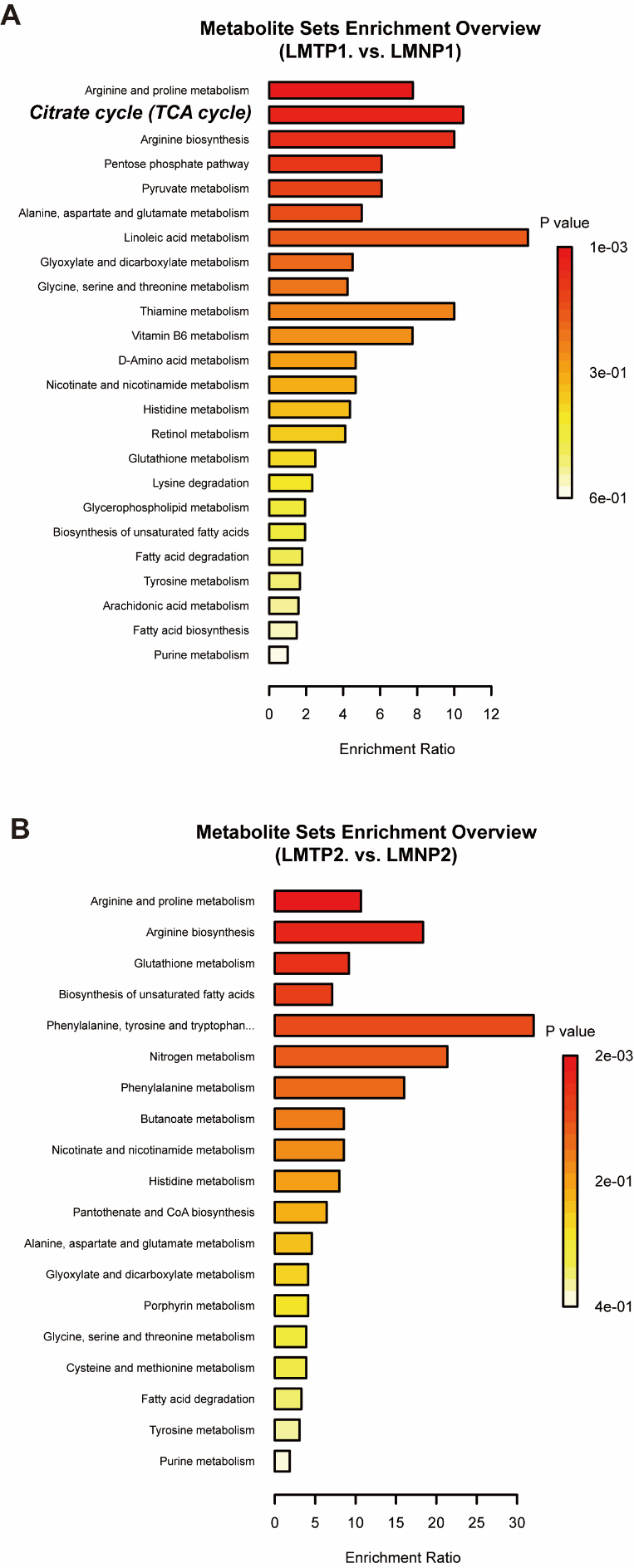
**

**Supplementary Figure 2. Enrichment analysis of differential metabolites between P1 or P2 liver metastases and normal liver.**

(A) KEGG pathway enrichment of the positive and negative ion metabolites of LMTP1 and LMNP1. (B) KEGG pathway enrichment of the positive and negative ion metabolites of LMTP2 and LMNP2.


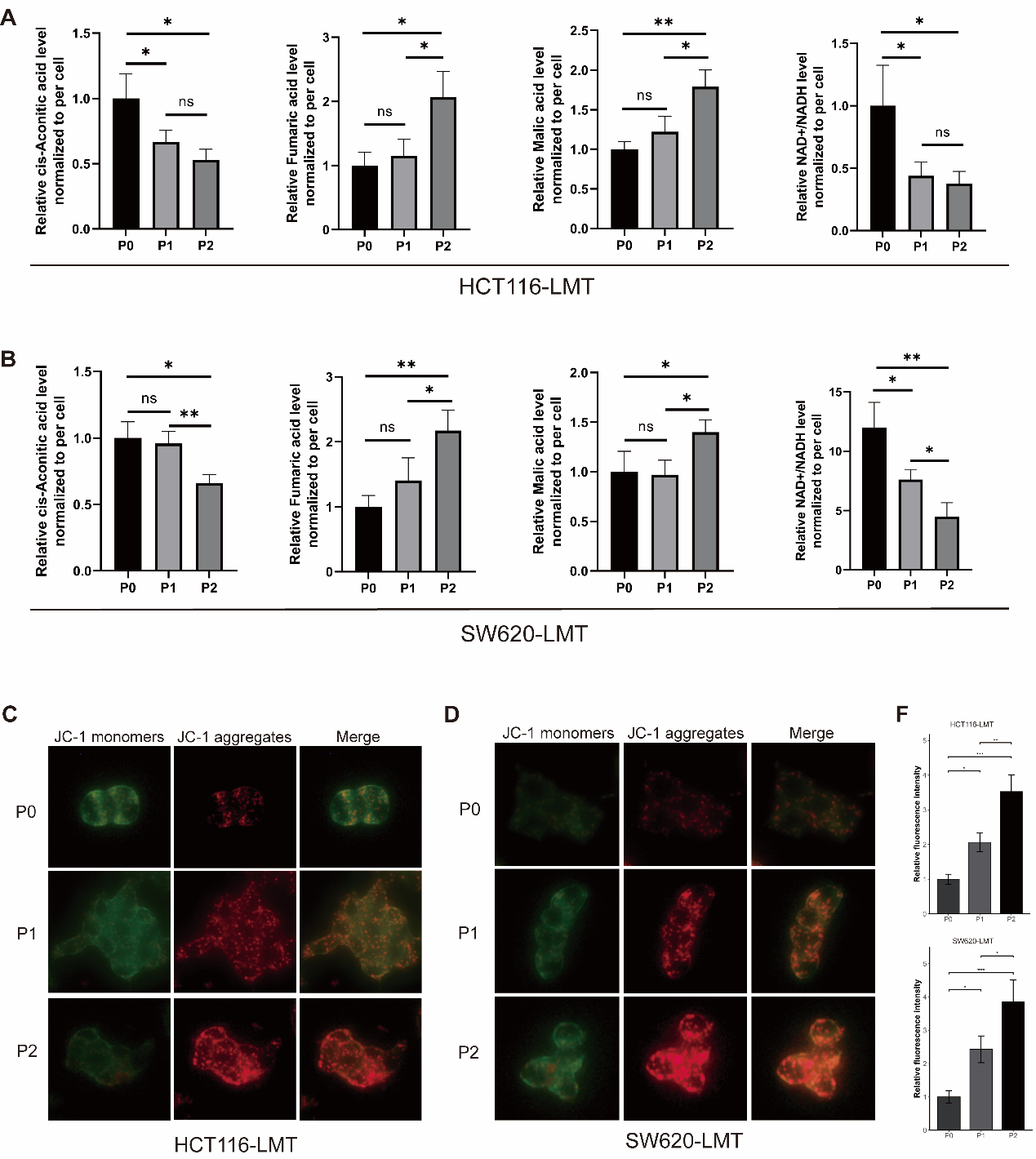


**Supplementary Figure 3. Metabolic alterations and mitochondrial membrane potential changes across different generations (P0, P1, P2) of HCT116-LMT and SW620-LMT cells.**

(A) Quantification of relative levels of Isocitric acid, Fumaric acid, Malic acid, and the NAD+/NADH ratio in HCT116-LMT cells at different generations (P0, P1, P2). (B) The corresponding metabolite levels in SW620-LMT cells. *p < 0.05, *p < 0.01, ns = not significant. (C) Representative fluorescence images of JC-1 staining in HCT116-LMT cells at different generations (P0, P1, P2). JC-1 monomers (green) indicate depolarized mitochondria, whereas JC-1 aggregates (red) represent mitochondria with intact membrane potential. The merged images illustrate the overall mitochondrial membrane potential (MMP) distribution. (D) Corresponding JC-1 staining images for SW620-LMT cells. (F) Quantitative Analysis of JC-1 Fluorescence Intensity Across Different Passages (P0, P1, P2) in HCT116 and SW620 Cell Lines.


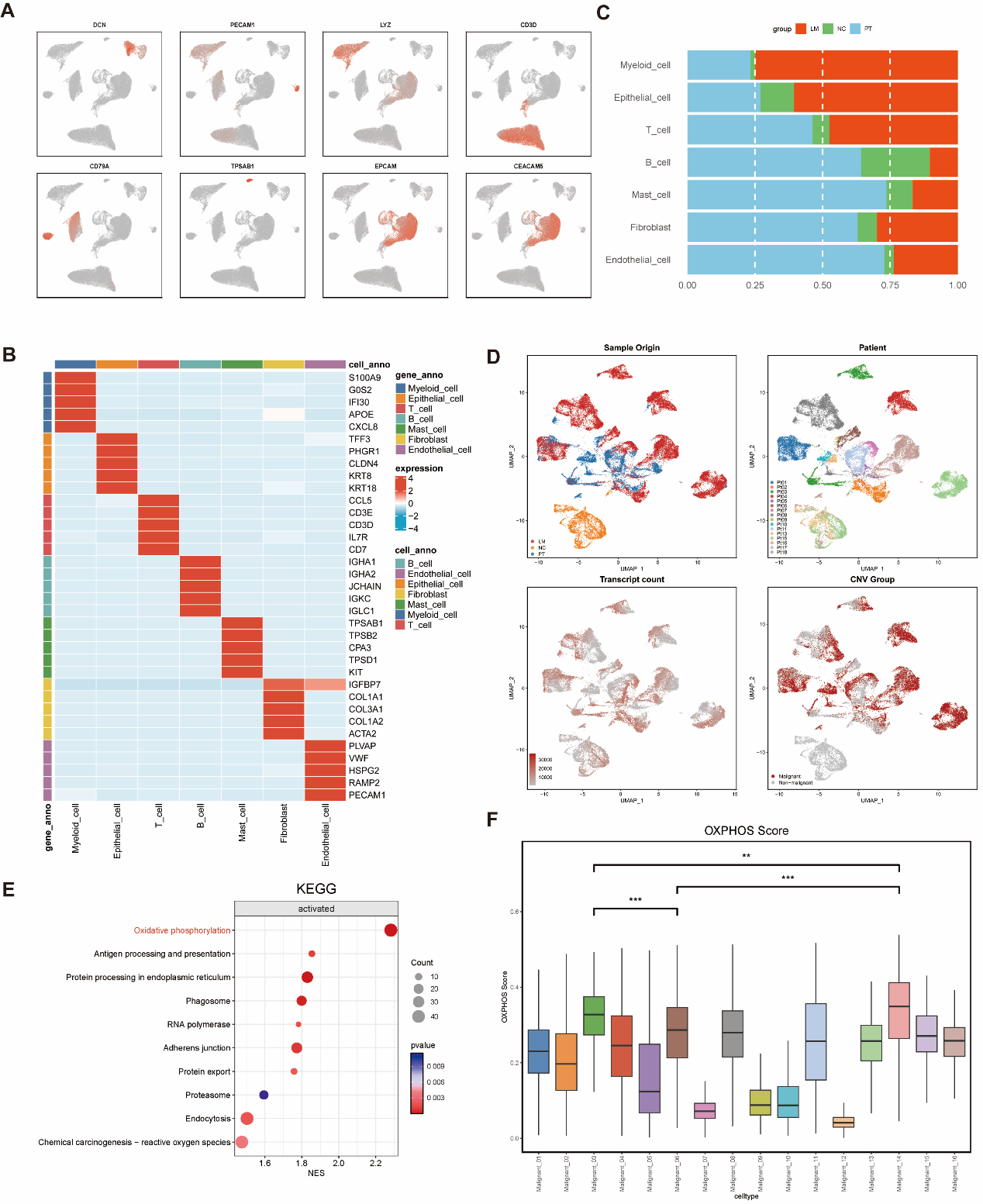


**Supplementary Figure 4. Overview of single-cell transcriptomic analysis of CRC samples.**

(A) The UMAP plot shows the distribution of classic marker genes for the seven cell types. (B) The heatmap presents the top 5 marker genes of seven cell types. (C) The proportion of each cell type in samples from different regions. (D) UMAP plots of all epithelial cells in this study were colored based on sample origin, patient, transcript count and CNV group. (E) The top 10 pathways enriched by KEGG based on the differential genes between LM and PT. (F) Boxplot of OXPHOS scores in Malignant Epithelial Cells.


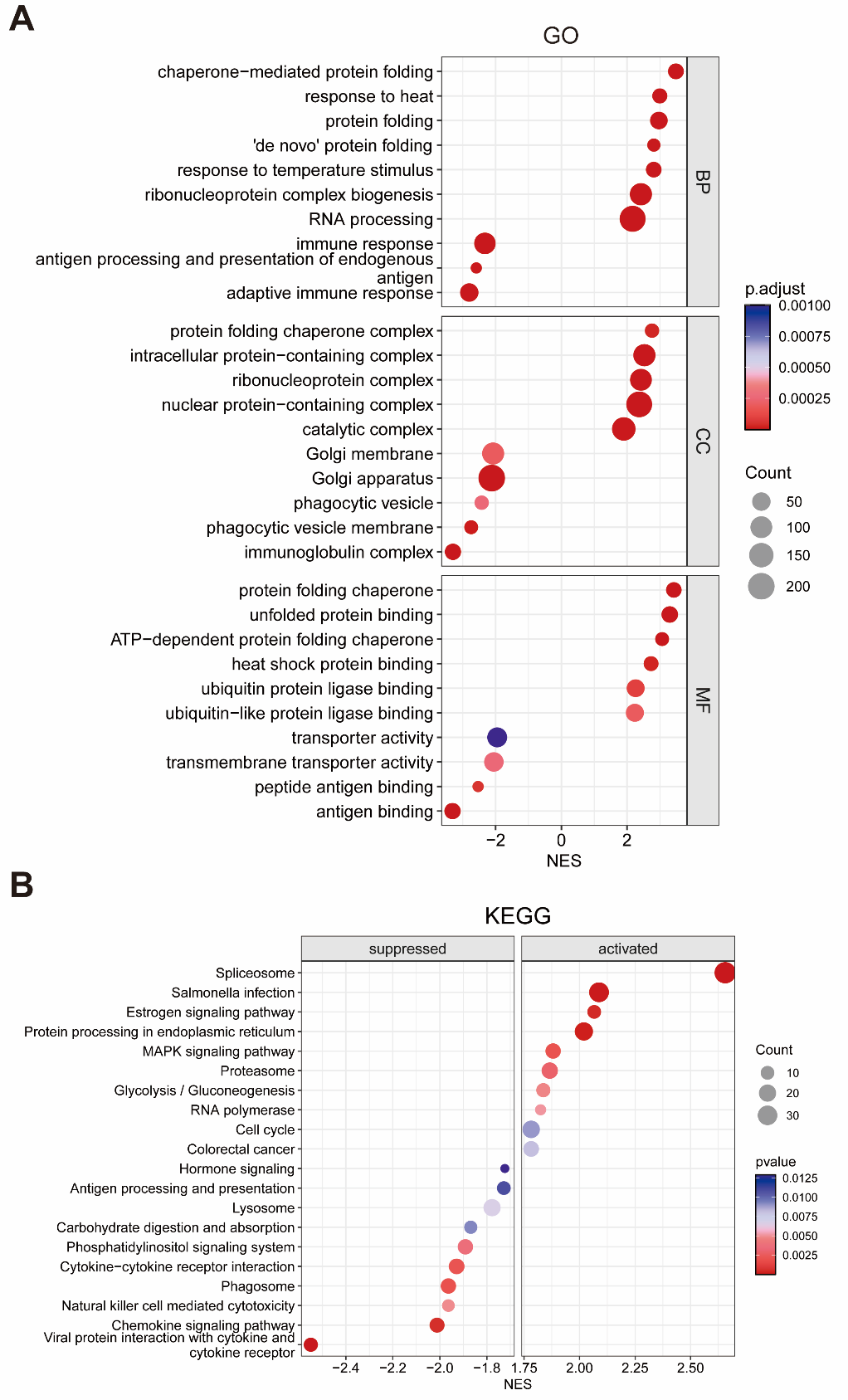


**Supplementary Figure 5. Enrichment analysis of differential pathways between Malignant_03 and Malignant_14.**

(A) The top ten pathways with the highest absolute values of NES in the BP, CC and MF categories in the GO enrichment analysis. (B) The top twenty pathways with the highest absolute values of NES in KEGG enrichment analysis.


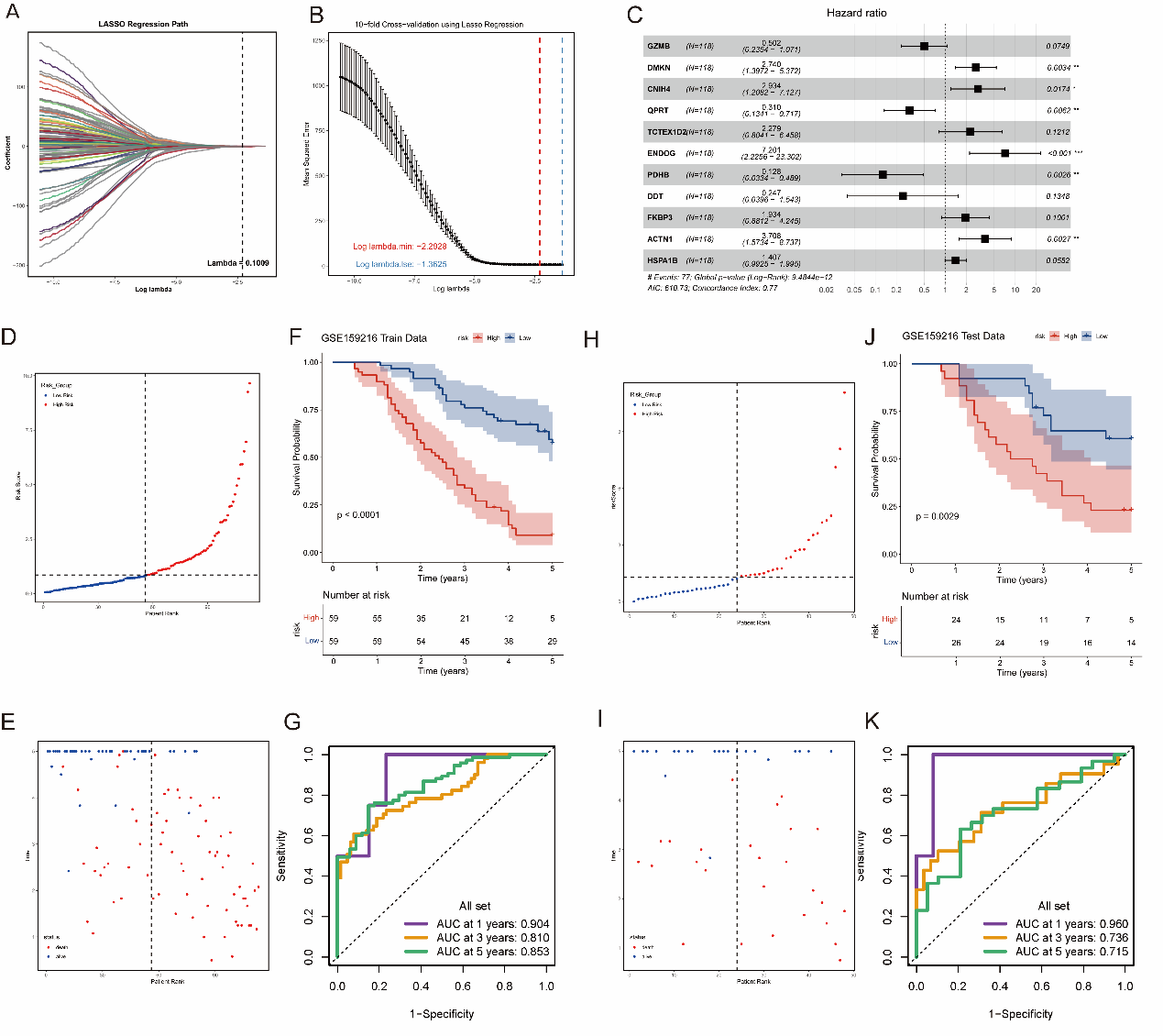


**Supplementary Figure 6. The model was constructed and validated using the differential genes from the Malignant_14.**

(A, B) Ten-fold cross-validation determined the penalty regularization parameter λ for gene selection. (C). Multivariate Cox regression analysis of the genes in the model. (D, E). The risk score and corresponding survival status of samples from the GSE159216 train dataset. (F). The relationship between the risk score of samples in the GSE159216 train dataset and OS. (G). The relationship between risk scores and ROC curves of samples from the GSE159216 train dataset. (H, I)The risk score and survival status of the ascending sorted samples in the GSE159216 test dataset. (J). The relationship between the risk score of samples in the GSE159216 train dataset and OS. (K). The relationship between risk scores and ROC curves of samples from the GSE159216 train dataset.


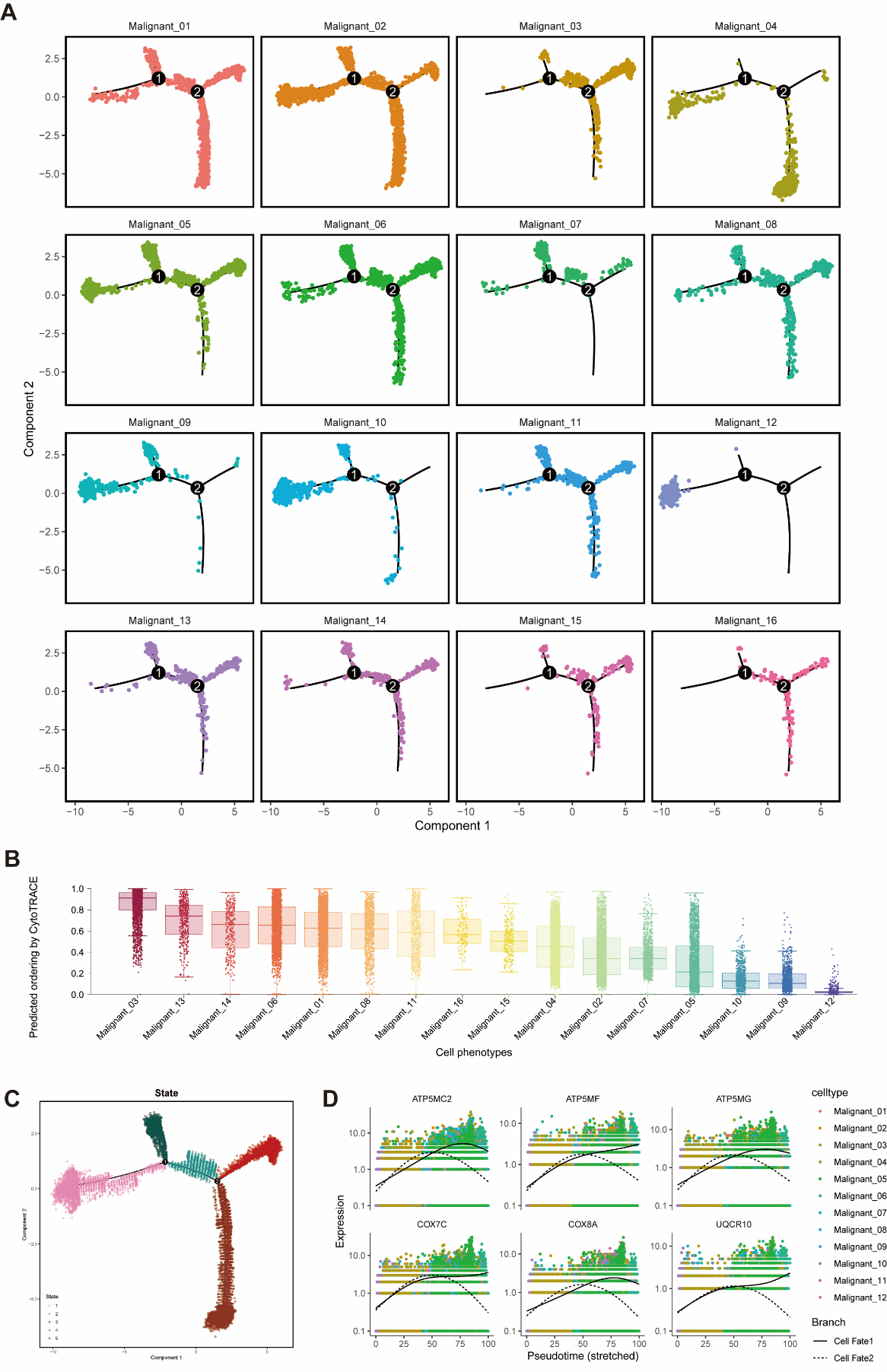


**Supplementary Figure 7. Analysis of epithelial cell subclusters and developmental trajectory.** (A) The trajectory plot illustrates the distribution of all malignant epithelial cell subclusters. (B) The boxplot illustrates the CytoTRACE scores of all malignant epithelial cell subclusters. (C) The potential trajectories of all malignant epithelial cells are distinctly colored according to the trajectories state. (D) The dot plot delineates the pseudotime trajectory of certain OXPHOS-related genes.
